# Heterogeneous progression of AT(N) biomarkers in data-driven subtypes of Alzheimer’s disease

**DOI:** 10.3389/fnagi.2026.1902282

**Published:** 2026-10-13

**Authors:** Zhiwei Teng, Mengyun Hu, Zhongwei Zhang, Mingqi Zhou, Ping Yin, the Alzheimer’s Disease Neuroimaging Initiative

**Affiliations:** 1 Department of Epidemiology and Biostatistics, School of Public Health, Tongji Medical College, Huazhong University of Science and Technology, Wuhan, China; 2 Department of Nursing, Union Hospital, Tongji Medical College, Huazhong University of Science and Technology, Wuhan, Hubei, China

**Keywords:** Alzheimer’s disease, APOE ε4, AT(N) biomarkers, disease subtypes, latent time, longitudinal joint model

## Abstract

**Background:**

Alzheimer’s disease (AD) exhibits substantial heterogeneity in brain atrophy patterns and progression rates. Data-driven subtyping based on the Subtype and Stage Inference (SuStaIn) model has identified distinct MRI-derived subtypes, but the longitudinal dynamics of these subtypes within the AT(N) framework remain unknown.

**Methods:**

This study included 1,576 participants from the Alzheimer’s Disease Neuroimaging Initiative (ADNI) with at least two visits. SuStaIn was applied to volumetric measurements of 11 brain regions to identify imaging-based subtypes. Within each subtype, we fitted a Bayesian latent time longitudinal joint mixed-effects model incorporating six biomarkers: amyloid PET (A), CSF p-tau (T), hippocampal volume, cingulate volume, thalamic volume (N), and ADAS-Cog13 (X), with APOE ε4 as a covariate.

**Results:**

The three subtypes exhibited distinctly different longitudinal patterns. Subtype 1 showed early elevation of amyloid, followed by a sharp decline in hippocampal volume, which was tightly coupled with cognitive deterioration. Subtype 2 featured early involvement of cortical regions such as the cingulate cortex, with hippocampal changes occurring at a similar timing to Subtype 1 but accompanied by earlier cortical and thalamic involvement. Subtype 3 exhibited early rapid hippocampal atrophy that later plateaued, and demonstrated the strongest hippocampus–cognition coupling. The association of APOE ε4 with biomarker trajectories was subtype-dependent: its effect estimates were largest for amyloid and p-tau in Subtype 1, whereas in Subtype 3 the effect estimates were significant for nearly all biomarkers, with the largest estimate for cognition.

**Conclusion:**

This study demonstrates that AD subtypes defined by spatial atrophy patterns also differ fundamentally in temporal biomarker dynamics and APOE ε4 association, supporting subtype-stratified modeling of disease progression.

## Introduction

Alzheimer’s disease (AD) is a neurodegenerative disorder characterized primarily by progressive cognitive impairment. The pathological process typically begins 10–20 years before the appearance of clinical symptoms, manifesting as the gradual accumulation of β-amyloid (Aβ) deposition and abnormal tau protein phosphorylation, ultimately leading to neuronal damage and brain structural atrophy (Jack Jr et al., 2013). This process has been encapsulated by the amyloid cascade hypothesis (Hardy and Higgins, 1992). AD is the most common form of dementia worldwide, with prevalence rising exponentially with age, imposing a heavy burden on both society and families (Alzheimers Dement, 2025; Scheltens et al., 2021). Although this prolonged evolution provides a potential window for early intervention, AD also exhibits marked heterogeneity: some patients predominantly present with episodic memory deficits, whereas others show language or visuospatial dysfunction; in terms of progression rate, there are also substantial individual differences ranging from slow evolution to rapid deterioration (Dubois et al., 2014). Nevertheless, this hypothesis faces challenges (Drachman, 2014); for instance, the degree of Aβ burden in the brain does not always match the severity of cognitive impairment in some patients. Recent anti-amyloid antibodies can reduce Aβ burden, but their benefits are limited in patients at middle or late stages (van Dyck et al., 2023; Sims et al., 2023). The AT(N) framework, which organizes AD biomarkers into amyloid deposition (A), tau pathology (T), and neurodegeneration (N), has provided a systematic approach to track disease progression along this pathological cascade (Jack Jr et al., 2010).

From a modeling perspective, the heterogeneity of AD can be further summarized along two core dimensions: spatial and temporal. In the spatial dimension, different patients may exhibit different patterns of regional brain involvement, such as a typical subtype predominantly affecting the medial temporal lobe or a variant type primarily involving cortical regions. In the temporal dimension, there are marked differences among individuals in disease onset time and progression rate; some patients progress slowly with a disease course lasting more than a decade, whereas others deteriorate rapidly over a relatively short period (Crutch et al., 2017; Holland et al., 2012). These features suggest that AD is not a single homogeneous disease but rather comprises multiple subtypes with different progression pathways. Therefore, relying solely on population-average trajectories is insufficient to accurately describe the disease progression process, and a modeling framework that can simultaneously capture subtype architecture and individual progression processes is required (Young et al., 2018).

Crucially, genetic factors, particularly the APOE ε4 allele, play a pivotal role in driving this heterogeneity. As the strongest genetic risk factor for late-onset AD, APOE ε4 is associated with earlier disease onset, accelerated cognitive decline, and distinct patterns of brain atrophy (Aksman et al., 2023). Recent evidence suggests that the influence of APOE ε4 on disease progression is not uniform but may vary across different AD subtypes, potentially modulating the trajectory of amyloid and tau pathology as well as neurodegeneration (Estarellas et al., 2024; Aksman et al., 2023). For instance, APOE ε4 carriers may exhibit more aggressive medial temporal lobe atrophy or faster cortical thinning depending on the underlying subtype architecture. Understanding these subtype-specific genetic influences is essential for unraveling the complex interplay between genetics and pathophysiology in AD.

Data-driven disease progression models offer new analytical tools to address the above challenges. Among these, the Subtype and Stage Inference (SuStaIn) model combines event-sequence modeling with clustering analysis to simultaneously identify disease subtypes and their progression stages within a unified framework (Young et al., 2018). This method has revealed imaging-based subtypes in AD, including the typical, cortical, and subcortical subtypes as originally defined by Young et al. (2018), substantially improving our ability to characterise spatial heterogeneity in the disease. However, the temporal characterisation in SuStaIn is still based on relative stages defined by event sequences and lacks the capacity to model a real-time scale; moreover, such methods typically do not explicitly handle the temporal misalignment across individuals during the disease course, thereby limiting a fine-grained description of the dynamic evolution of the disease (Oxtoby et al., 2017; Raj et al., 2012).

To overcome these limitations, researchers have developed longitudinal joint models that align individual observations onto a common latent disease time axis (Li et al., 2018; Lorenzi et al., 2019). By estimating individual-specific latent time shifts, these models can integrate heterogeneous longitudinal data and reconstruct continuous trajectories of biomarkers as the disease advances, thus achieving population-level temporal alignment while preserving individual differences.

Against this background, the present study integrates SuStaIn subtyping with a latent-time longitudinal joint mixed-effects model to systematically analyse the longitudinal progression patterns of AT(N) biomarkers across different AD subtypes in the Alzheimer’s Disease Neuroimaging Initiative (ADNI) cohort. Specifically, we first identified imaging-based subtypes of AD using volumetric data from structural magnetic resonance imaging (MRI). Subsequently, within each subtype, we constructed a latent-time longitudinal model to characterise the trajectories of amyloid PET (A), CSF p-tau (T), hippocampal volume, cingulate volume, thalamic volume (N), and ADAS-Cog13 (X) as disease progresses. Furthermore, we explored whether APOE ε4, the strongest genetic risk factor, is associated with disease progression in a subtype-dependent manner, aiming to provide a basis for subtype-stratified and individualised prognostic assessment of AD.

## Methods

### Study population and data source

Data were obtained from the ADNI database.^1^ ADNI is a longitudinal, multi-center observational study initiated in 2003 (Petersen et al., 2010). Inclusion criteria were: (1) at least two follow-up visits; (2) complete information on age and APOE ε4 genotype; and (3) at least one measurement of the six target biomarkers (amyloid PET, CSF p-tau, hippocampal volume, cingulate volume, thalamic volume, ADAS-Cog13). Sample sizes for each of the six longitudinal biomarkers stratified by baseline diagnosis are summarized in Supplementary Table S4. The three MRI structural markers (hippocampal volume, cingulate volume, and thalamic volume) were available for all 1,576 participants, providing complete foundational data for SuStaIn subtyping. Amyloid PET and CSF p-tau had partial missingness (76.8 and 76.3% baseline availability, respectively), which was accommodated by the Bayesian joint model’s natural handling of missing data (Section 2.4). All 11 MRI-based volumetric features required for SuStaIn subtyping were available for all participants included in this study. After applying the above inclusion and exclusion criteria, a total of 1,576 participants were included in the longitudinal analysis. Based on the SuStaIn subtyping results, the subtype distribution was: Subtype 1, *n* = 625; Subtype 2, *n* = 592; Subtype 3, *n* = 359. Data used in this study were obtained from the Alzheimer’s Disease Neuroimaging Initiative (ADNI) database (see footnote 1). ADNI was launched in 2003 as a public-private partnership, led by Principal Investigator Michael W. Weiner, MD, VA Medical Center and University of California, San Francisco. The primary goal of ADNI has been to test whether serial magnetic resonance imaging (MRI), positron emission tomography (PET), other biological markers, and clinical and neuropsychological assessment can be combined to measure the progression of mild cognitive impairment (MCI) and early Alzheimer’s disease (AD). For up-to-date information, see www.adni-info.org. The investigators within the ADNI contributed to the design and implementation of ADNI and/or provided data but did not participate in analysis or writing of this report.

### MRI processing and SuStaIn-based subtype identification

T1 weighted structural MRI data from all participants were acquired using 3.0 Tesla scanners. Cortical reconstruction and subcortical segmentation were performed using FreeSurfer v6.0, with cortical regions parcellated according to the Desikan–Killiany atlas (via the aparc.stats output) and subcortical structures segmented using the standard aseg.stats segmentation pipeline. This yielded grey matter volumes for 11 regions of interest (ROIs): frontal lobe, temporal lobe, parietal lobe, occipital lobe, cingulate gyrus, insula, hippocampus, amygdala, thalamus, nucleus accumbens, and caudate nucleus. Notably, the four lobar ROIs (frontal, temporal, parietal, occipital) are not native outputs of the FreeSurfer parcellation; they were constructed by aggregating (summing) the volumes of their respective constituent gyral subregions defined by the Desikan–Killiany atlas. The complete subregion-to-lobe mapping used for this aggregation is included in Supplementary Table S1 (columns 2 and 3 list the FreeSurfer region codes and their corresponding anatomical region labels, respectively). To eliminate the effects of inter individual differences in head size and age on volume measurements, a multivariate linear regression model of each ROI volume against age and intracranial total volume (ICV) was established based on cognitively normal, amyloid negative healthy controls(cognitively normal was defined as baseline MMSE ≥ 27, CDR = 0, and no subjective or objective memory complaints confirmed by study physicians; amyloid-negative was defined using ADNI standardized Florbetapir PET SUVr with a threshold of <1.11, referenced to the cerebellar cortex), and covariate adjusted z scores were calculated (Lorenzi et al., 2019). The specific calculation is given in Equation (1):


$$z=\frac{y-{\hat{y}}_{\text{healthy}}}{{\mathrm{SD}}_{\text{residual}}},$$ (1)

where y is the individual ROI volume, ${\hat{y}}_{\text{healthy}}$ is the value predicted from the healthy control group, and ${\mathrm{SD}}_{\text{residual}}$ is the standard deviation of the residuals in the healthy control group. Because neurodegenerative atrophy results in negative raw z-scores (smaller values indicate more severe atrophy), and SuStaIn requires that higher input values represent greater disease severity, all z-scores were reversed in sign (multiplied by −1) so that increasing values reflect increasing abnormality.

The SuStaIn algorithm (Young et al., 2018) was applied to the reversed z-scores of the 11 ROIs for subtype and stage inference. Three increasing abnormality thresholds were preset for each ROI (0.5, 1.5, and 2.5 standard deviations for most regions; 1.0, 2.0, and 3.0 for hippocampus and amygdala due to their smaller baseline variability), generating a total of 33 discrete events. The model employed Markov Chain Monte Carlo (MCMC) sampling to estimate the posterior event sequences. The cross-validation information criterion (CVIC) and log-likelihood for models with 1 to 4 subtypes were compared (Supplementary Figure S1).

### Standardisation of biomarkers for longitudinal modelling

Biomarkers used for longitudinal modelling also suffer from differences in units and distributional shapes. To compare the severity of different biomarkers on a common scale, this study adopted a rank-based standardisation method that transforms raw biomarker values into severity metrics on a standard normal scale. This method first obtains the quantile position of each observation in the overall distribution using a weighted empirical cumulative distribution function (weighted ECDF), and then maps it to the standard normal space via a normal quantile transformation.

Let $y_{\mathrm{ib}}$ denote the raw observation of the b -th biomarker for the i -th subject, and $d_{i}$ denote the diagnostic group (e.g., normal cognition, mild cognitive impairment, or Alzheimer’s disease). Because sample sizes may be unbalanced across diagnostic groups, to prevent any single group from exerting undue influence on the overall distribution, each diagnostic group was assigned equal weight. Specifically, if the g -th diagnostic group contains $n_{g}$ samples, the weight for each observation in that group is defined by Equation (2):


$$w_{\mathrm{ig}}=\frac{1}{n_{g.}}$$ (2)

Based on this, the weighted empirical cumulative distribution function is constructed as shown in Equation (3):


$${\hat{F}}_{b}(x)=\frac{\sum_{j=1}^{N}w_{j}1(x_{\mathrm{jb}}\leq x)}{\sum_{j=1}^{N}w_{j}}$$ (3)

where 1(·) is the indicator function, equal to 1 if the condition holds and 0 otherwise; ${\hat{F}}_{b}(x)$ represents the quantile position of observation x in the weighted overall distribution.

Subsequently, this quantile is transformed via the inverse cumulative distribution function of the standard normal distribution to obtain the biomarker severity Z-score, as shown in Equation (4):


$$z_{\mathrm{ib}}=\Phi^{-1}({\hat{F}}_{b}(x_{\mathrm{ib}}))$$ (4)

where $\Phi^{-1}(\cdot )$ is the inverse cumulative distribution function of the standard normal distribution.

To avoid infinite values arising from $\Phi^{-1}(0)$ or $\Phi^{-1}(1)$ during numerical computation, the quantile was truncated according to Equation (5):


$$u_{\mathrm{ib}}=\min(\max({\hat{F}}_{b}(x_{\mathrm{ib}}),\varepsilon ),1-\varepsilon ),z_{\mathrm{ib}}=\Phi^{-1}(u_{\mathrm{ib}})$$ (5)

where ε is a small constant (e.g., $10^{-4}$ ).

After the above transformation, each biomarker is mapped to an approximately standard normal scale, making different biomarkers comparable while preserving the monotonic ordering of the original data. The transformed $z_{\mathrm{ib}}$ can be interpreted as the standardised position of that observation in the overall disease severity distribution, and serves as the input variable for subsequent longitudinal disease progression modelling.

### Latent-time longitudinal joint mixed-effects model

Within each subtype, we fitted a Bayesian joint model to analyse six longitudinal biomarkers: amyloid PET (A), CSF p-tau (T), hippocampal volume, cingulate volume, thalamic volume (N), and ADAS-Cog13 (X). The model incorporates subject-specific latent time shifts to align individual progression onto a common timescale.

Let $y_{\mathrm{ib}}(t_{\mathrm{ij}})$ denote the standardised value of biomarker b for subject i at calendar time $t_{\mathrm{ij}}$ (age). The model is specified in Equation (6):


$$y_{\mathrm{ib}}(t_{\mathrm{ij}})=\beta_{b}+\gamma_{b}(t_{\mathrm{ij}}+\lambda_{i})+\alpha_{0\mathrm{ib}}+\alpha_{1\mathrm{ib}}t_{\mathrm{ij}}+\in_{\mathrm{ibj}},$$ (6)

where:

$\beta_{b}$ is the fixed intercept for biomarker b ;

$\gamma_{b}>0$ is the population-level progression rate (slope on latent time);

$\lambda_{i}∼N(0,\sigma_{\lambda }^{2})$ is the subject-specific latent time shift;

$\alpha_{0\mathrm{ib}},\alpha_{1\mathrm{ib}}$ are the random intercept and random slope for subject i and biomarker b ;

$\in_{\mathrm{ibj}}∼N(0,\sigma_{b}^{2})$ is the residual error.

To ensure identifiability between $\lambda_{i}$ and $\alpha_{0\mathrm{ib}}$ , a zero-sum constraint was imposed: for each subject, the sum of random intercepts across all biomarkers is zero, i.e., $\sum_{b=1}^{6}\alpha_{0\mathrm{ib}}=0$ . APOE ε4 genotype was included as a covariate in $\beta_{b}$ .

In selecting the cortical marker for the “N” dimension, the present study chose cingulate volume over parietal or temporal cortical volumes based on the following considerations: First, the goal of longitudinal modeling is to compare temporal progression differences across subtypes; therefore, it is necessary to select a region that exhibits observable changes across all subtypes, rather than a region that is definitional for a specific subtype. Second, the posterior cingulate cortex is a well-recognized early cortical involvement region in the AD course, and its cross-phenotype high sensitivity has been repeatedly demonstrated in studies of default mode network degeneration and amyloid deposition (Scheff et al., 2015; Lee et al., 2020). Third, the data from the present study show relatively high correlations among cortical regions (*r* ≈ 0.4–0.6), and including multiple cortical regions could introduce collinearity issues. As a hub region connecting the medial temporal lobe with the parietal–frontal association cortex, the cingulate cortex possesses both limbic and association cortex features in functional anatomy, making it a suitable single cortical degeneration indicator.

### Prior specification and parameter estimation

Weakly informative priors were used: $\beta_{b},\gamma_{b}∼N(0,100)$ (with $\gamma_{b}$ truncated to be positive); $\sigma_{\lambda },\sigma_{b},\sigma_{\alpha 0},\sigma_{\alpha 1}∼\text{Half}-\text{Cauchy}(0,2.5)$ ; the correlation matrix of random effects was assigned an LKJ(2) prior. The model was implemented using the NUTS sampler in Stan (version 2.26). For each subtype, four chains were run, each with 2,000 iterations (the first 1,000 as warm-up), yielding a total of 4,000 posterior samples. Convergence was assessed by $\hat{R}<1.05$ and visual inspection of trace plots (illustrated for subtype 3 in Supplementary Figure S1).

### Statistical analysis

Posterior means and 95% credible intervals (CrI) were reported for all parameters. A difference between two subtypes was considered significant if the 95% CrIs of their $\gamma_{b}$ did not overlap. The distributions of latent time shifts ( $\lambda_{i}$ ) were compared across different diagnostic groups and APOE ε4 status. Model fit was evaluated using scatterplots of observed versus predicted values (Supplementary Figures S3–S5).

To validate the impact of the cortical “N” metric selection on longitudinal modeling results, we conducted a sensitivity analysis by replacing the cingulate volume with the parietal volume and refitting the latent time model for Subtype 2. The parietal volume was defined as the sum of the superior parietal and inferior parietal cortical regions according to the Desikan–Killiany atlas. Results of this sensitivity analysis are provided in Supplementary Table S2.

### External validation in an independent Chinese cohort

To preliminarily evaluate the generalizability of our subtype-stratified progression model to non-Caucasian populations, we conducted an exploratory external validation in a small independent Chinese cohort (*N* = 10) recruited from the Department of Psychiatry, Sir Run Run Shaw Hospital, Zhejiang University School of Medicine. The study protocol was approved by the hospital’s ethics committee. All patients met the same inclusion criteria as the ADNI cohort: (1) diagnosis of Alzheimer’s disease or cognitive impairment; (2) at least two follow-up visits; (3) complete APOE genotyping; and (4) structural T1-weighted 3 T MRI data passing FreeSurfer quality control. The same MRI processing pipeline (FreeSurfer v6.0, Desikan–Killiany atlas), z-score standardization, and SuStaIn subtyping procedures were applied as described in Sections 2.2 and 2.3. The pre-trained ADNI models were used to assign subtype labels and predict individual biomarker trajectories. Predicted trajectories were compared with observed values to assess model performance in this independent dataset.

## Results

### Participant characteristics and subtype distribution

The three subtypes differed significantly in demographic and clinical characteristics (Table 1). Age (*F* = 13.42, *p* < 0.001), sex (*χ*^2^ = 20.79, *p* < 0.001), and APOE ε4 carrier status (*χ*^2^ = 23.61, *p* < 0.001) all differed significantly across subtypes, whereas the number of follow-up visits did not (*F* = 1.07, *p* = 0.343). Subtype 3 was the oldest (mean 73.3 years) and had the highest proportion of males (59.6%) and APOE ε4 carriers (52.1%), while Subtype 2 was the youngest (mean 70.7 years) and had the lowest APOE ε4 carrier rate (36.8%). Subtype 3 had the oldest age (mean 73.3 years), the highest proportion of males (59.6%), and the highest APOE ε4 carrier rate (52.1%). Subtype 2 was the youngest and had the lowest APOE ε4 carrier rate (36.8%). The number of follow-up visits was similar across subtypes (approximately 6 on average).

**Table 1:** Demographic characteristics of the three subtypes.

Characteristic	Subtype 1 (*N* = 625)	Subtype 2 (*N* = 592)	Subtype 3 (*N* = 359)	*p*-value
Age				<0.001
Mean (SD)	71.8 (7.4)	70.7 (7.0)	73.3 (6.7)	
Median [Min, Max]	72.0 [50.0, 90.0]	71.0 [55.0, 90.0]	73.0 [55.0, 90.0]	
Sex				<0.001
Male *N* (%)	279 (44.6)	299 (50.5)	214 (59.6)	
Female *N* (%)	346 (55.4)	293 (49.5)	145 (40.4)	
APOE ε4				<0.001
Carrier *N* (%)	271 (43.4)	218 (36.8%)	187 (52.1)	
Non-carrier *N* (%)	352 (56.3)	372 (62.8%)	172 (47.9)	
Number of visits				0.343
Mean (SD)	6.03 (3.5)	5.8 (3.4)	6.13 (3.5)	
Median [Min, Max]	5 [2, 20]	5 [2, 18]	5 [2, 21]	

APOE ε4 = apolipoprotein E ε4 allele. SD = standard deviation. APOE ε4 = apolipoprotein E ε4 allele. SD = standard deviation. *p*-values were derived from one-way ANOVA for continuous variables (age, number of visits) and chi-square tests for categorical variables (sex, APOE ε4 carrier status). Percentages may not sum to 100% due to rounding or missing data.

### Subtype-specific event sequences from cross-sectional SuStaIn

Figure 1 shows the Positional Variance Diagram (PVD) for the three subtypes based on cross-sectional data. Subtype 1 showed concurrent early involvement of subcortical structures (thalamus and nucleus accumbens) and medial temporal lobe structures (hippocampus and amygdala) at the earliest stages. The subsequent sequence extended to the temporal lobe and cingulate gyrus, and then to widespread association cortices and subcortical structures – broadly consistent with Braak staging (Braak and Braak, 1991; Lee et al., 2020), although the early thalamic/accumbens signals suggest additional subcortical involvement beyond the classical limbic-onset trajectory. Subtype 2 exhibited early involvement of frontal, parietal and cingulate regions, with relatively late hippocampal abnormalities, in line with the hippocampus-sparing variant described by Murray et al. (2011). Subtype 3 presented early events in the nucleus accumbens and caudate nucleus, followed later by hippocampal and cortical involvement, suggesting possible mixed pathology or network-based propagation mechanisms. To assess the robustness of the subtype structures, we additionally generated a PVD using pooled longitudinal data, which yielded consistent subtype ordering (Supplementary Figure S6).

**Figure 1:**
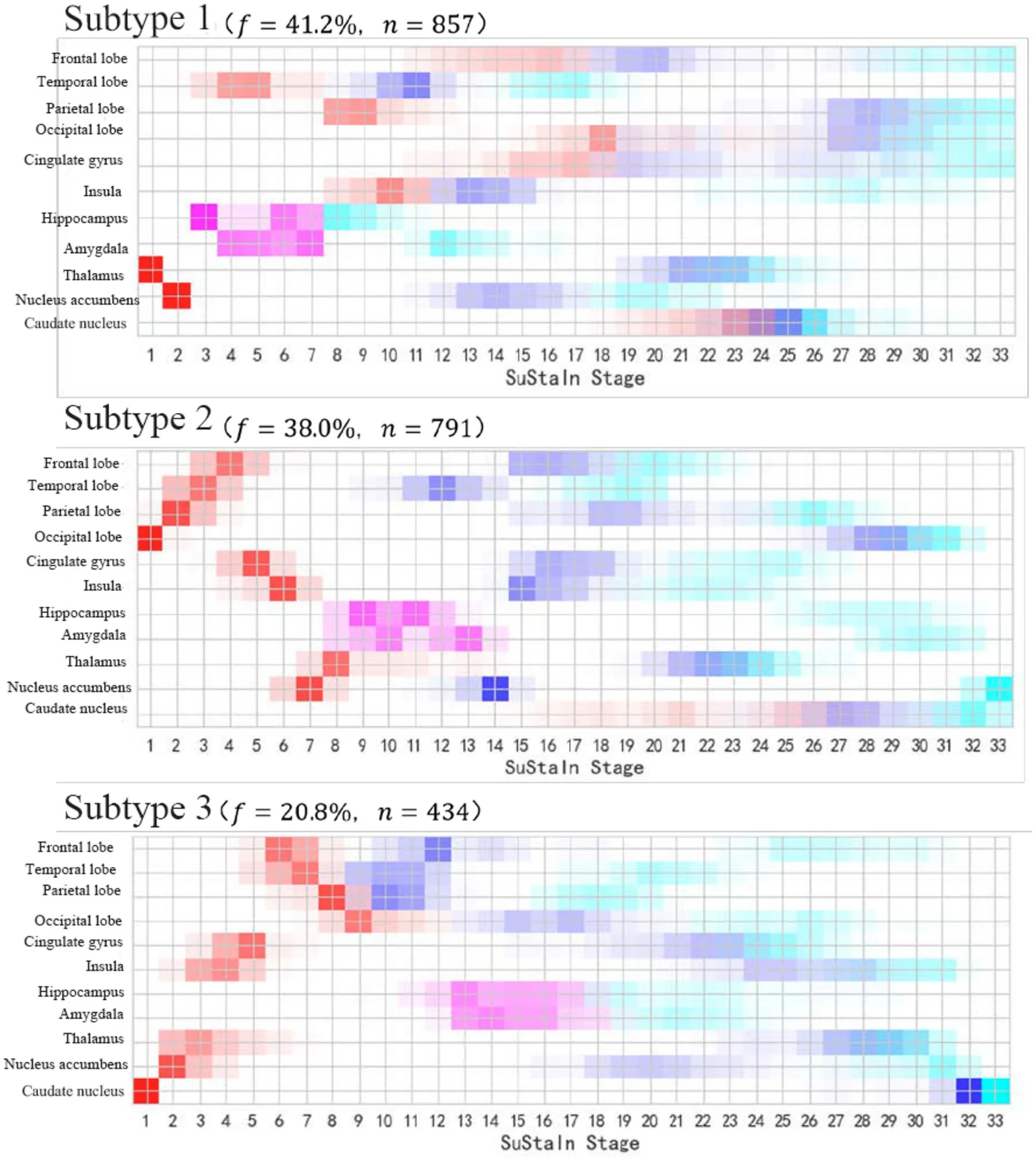
Cross-sectional positional variance diagram (PVD) for the three AD subtypes based on baseline MRI data. Each row represents a *z*-score event (brain region and threshold), and columns represent disease stages ordered from early (left) to late (right). Color intensity indicates the posterior probability of an event occurring at a given stage, ranging from white (probability 0) to red, magenta, and blue (highest probability). This visualization shows the most likely sequence of regional involvement for each subtype.

### Model convergence and latent time distribution

All parameters converged ( $\hat{R}<1.05$ ). Trace plots showed good mixing (Supplementary Figure S2). The estimated latent time shifts ( $\lambda_{i}$ ) progressively increased from cognitively normal to MCI to AD (Figure 2A), confirming that $\lambda_{i}$ reflects disease severity. No significant differences in $\lambda_{i}$ were observed by sex or APOE ε4 status (Figures 2B,C).

The latent-time-aligned age trajectories of the six biomarkers across diagnostic groups are shown in Figure 3.

**Figure 2:**
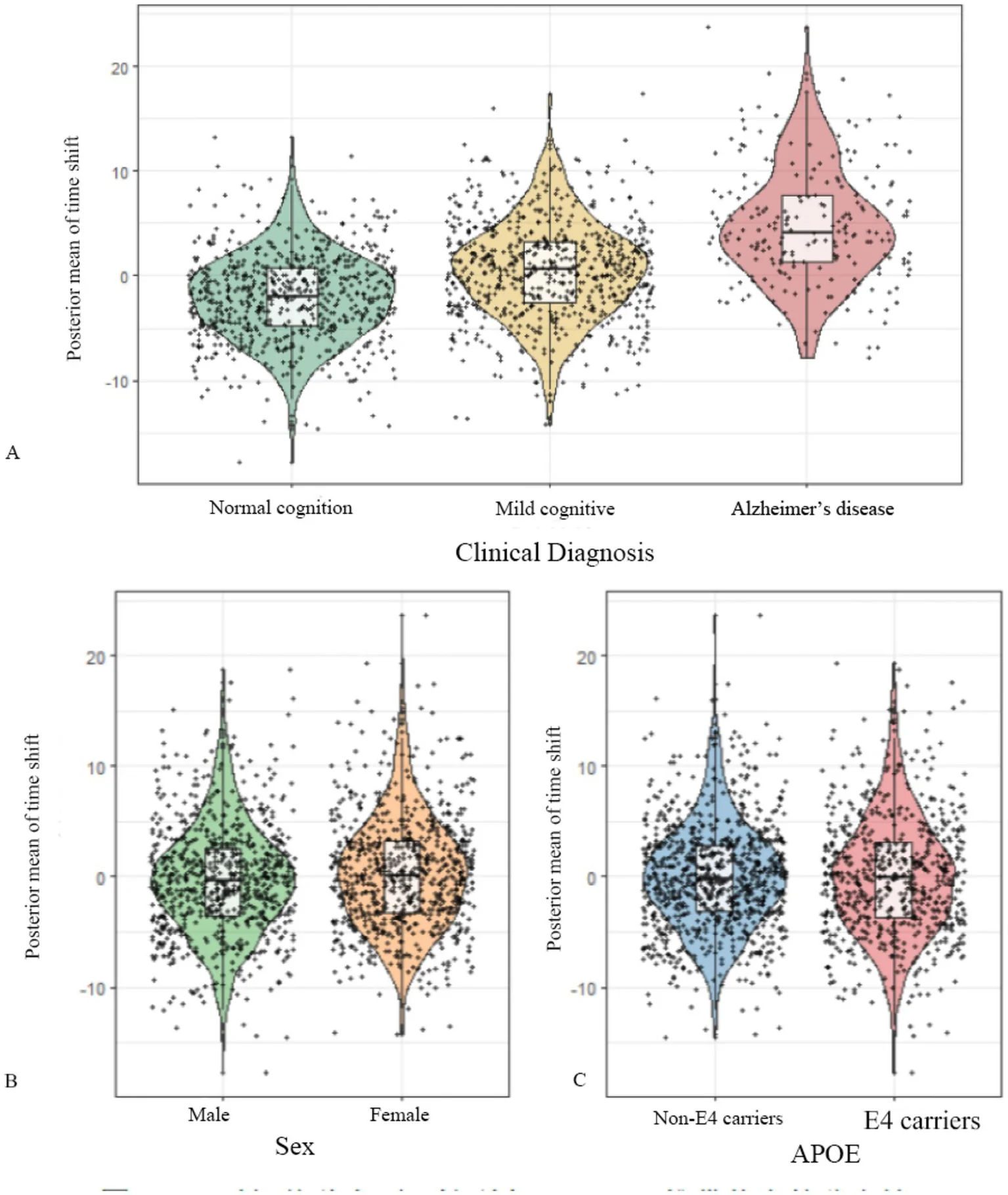
Distribution of estimated latent time shifts ( $\lambda_{i}$ ). **(A)** By diagnostic group (CN, MCI, AD). **(B)** By sex (male, female). **(C)** By APOE ε4 status (non-carrier, carrier). Boxplots show posterior means; $\lambda_{i}$ increases with disease severity but does not differ by sex or APOE ε4 status.

**Figure 3:**
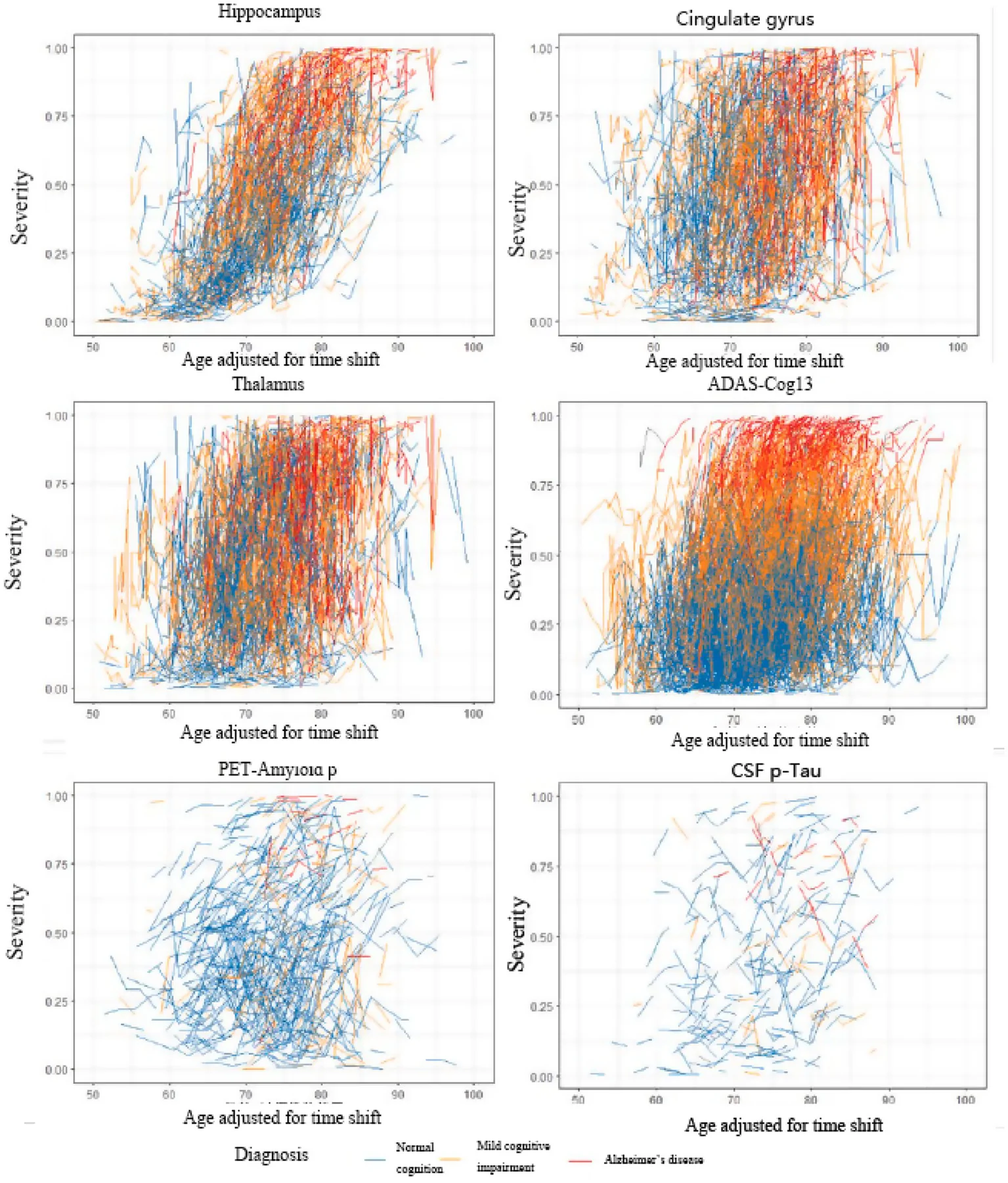
Corrected age trajectories of AT(N) biomarkers and cognition. Blue, orange, and red lines represent cognitively normal (CN), mild cognitive impairment (MCI), and Alzheimer’s disease (AD) groups, respectively. After alignment by latent time shifts, all biomarkers show clear monotonic progression patterns across diagnostic groups, in contrast to the uncorrected trajectories shown in Supplementary Figure S7.

### Subtype-specific longitudinal trajectories of AT(N) biomarkers

To examine whether APOE ε4 modulates the subtype-specific progression patterns, we stratified the predicted trajectories by APOE ε4 carrier status (Figures 4A,B). Figures 4A,B display the predicted progression curves for each biomarker on the latent disease time axis for APOE ε4 carriers (4A) and non-carriers (4B), respectively. The three subtypes exhibited distinctly different patterns: Subtype 1: Amyloid PET elevation occurred earliest (reaching ~0.35 at age 50 on the latent time axis), then slowly increased to ~0.8. Hippocampal volume started near normal (~0.05) but increased sharply after approximately age 70 (i.e., rapid atrophy), eventually reaching 0.95, and was tightly coupled with ADAS-Cog13 worsening. CSF p-tau rose linearly from a moderate level (~0.25). This pattern aligns with the classical amyloid cascade hypothesis, in which amyloid deposition precedes tau pathology, which in turn precedes neurodegeneration. Subtype 2: Both Subtype 1 and Subtype 2 showed similar early hippocampal involvement (Table 2; hippocampus progression slope (*γ*): 0.064 vs. 0.066). However, Subtype 2 showed early cortical involvement (cingulate gyrus), though the trajectory pattern was broadly similar to that of Subtype 1 in terms of timing of deviation, with a relatively higher cortical-to-subcortical slope ratio (progression slopes: 0.030/0.025 = 1.20 for Subtype 2 vs. 0.028/0.039 = 0.72 for Subtype 1; Table 2), suggesting that subcortical pathology coexists with the cortical-predominant pattern. Cognitive decline was initially slow, accelerated in the mid-to-late stages, and lagged behind cortical structural changes. This pattern supports a cortex-prioritized progression. However, the hippocampal trajectory of Subtype 2 was quantitatively similar to that of Subtype 1 (*γ* = 0.064 vs. 0.066; Table 2), and we therefore no longer characterize Subtype 2 as ‘hippocampal-sparing.’ Instead, Subtype 2 is distinguished primarily by its relatively higher cortical-to-subcortical slope ratio (cingulate/thalamus: 1.20 vs. 0.72 in Subtype 1). Subtype 3: Hippocampal volume showed very early and rapid deterioration, quickly reaching a high-severity plateau. Cognitive decline (ADAS-Cog13) was highly coupled with hippocampal loss. Amyloid and p-tau also increased but more gradually. Of note, the longitudinal model revealed that hippocampal atrophy dominated the dynamics for Subtype 3, suggesting a ‘subcortical-initiated—limbic-driven’ pattern.

**Figure 4:**
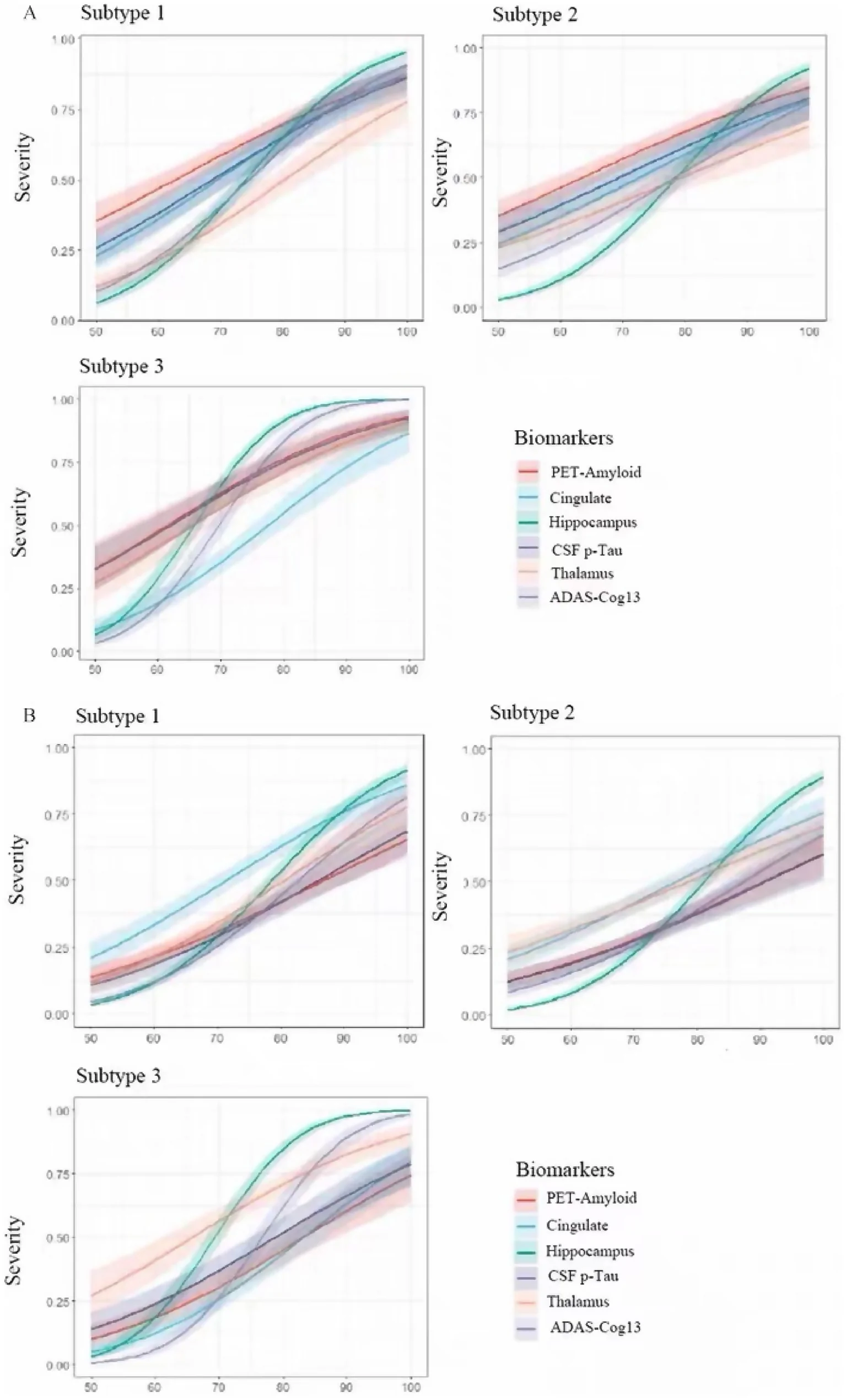
Predicted trajectories of AT(N) biomarkers across subtypes, stratified by APOE ε4 status: **(A)** carriers, **(B)** non-carriers. Subtype 3 shows early rapid hippocampal atrophy and strong cognitive coupling; Subtype 1 shows early amyloid then late hippocampal decline; Subtype 2 shows early cortical involvement with relatively later hippocampal changes, though the distinction from Subtype 1 is subtle. In Subtype 3, APOE ε4 effects are significant for all biomarkers except thalamus, with the largest effect on cognition, as reflected by the separation between carrier and non-carrier trajectories.

**Table 2:** Posterior estimates of progression slopes and APOE ε4 effects.

Biomarker	Posterior mean (95% CI)		
	Subtype 1	Subtype 2	Subtype 3
Amyloid PET			
Intercept	−2.583 (−2.952, −2.202)	−2.540 (−2.93, −2.155)	−3.222 (−3.839, −2.598)
APOE ε4 carrier	0.713 (0.603, 0.824)	0.756 (0.645, 0.869)	0.841 (0.66, 1.013)
Progression slope (*γ*)	0.030 (0.025, 0.035)	0.028 (0.023, 0.033)	0.039 (0.031, 0.047)
Residual	0.263 (0.250, 0.278)	0.25 (0.238, 0.262)	0.310 (0.290, 0.331)
Cingulate			
Intercept	−2.692 (−3.126, −2.264)	−2.331 (−2.715, −1.927)	−4.127 (−4.726, −3.491)
APOE ε4 carrier	0.068 (−0.062, 0.199)	0.122 (−0.020, 0.264)	0.281 (0.113, 0.44)
Progression slope (*γ*)	0.038 (0.032, 0.043)	0.030 (0.025, 0.036)	0.05 (0.041, 0.058)
Residual	0.307 (0.298, 0.317)	0.286 (0.277, 0.295)	0.394 (0.378, 0.410)
Hippocampus			
Intercept	−5.050 (−5.371, −4.742)	−5.388 (−5.694, −5.081)	−6.663 (−7.162, −6.154)
APOE ε4 carrier	0.294 (0.184, 0.402)	0.162 (0.051, 0.278)	0.340 (0.193, 0.483)
Progression slope (*γ*)	0.064 (0.060, 0.068)	0.066 (0.062, 0.071)	0.096 (0.089, 0.103)
Residual	0.22 (0.213, 0.228)	0.234 (0.227, 0.242)	0.228 (0.218, 0.238)
CSF *p*-tau			
Intercept	−2.978 (−3.435, −2.534)	−2.579 (−3.043, −2.122)	−2.986 (−3.575, −2.421)
APOE ε4 carrier	0.596 (0.446, 0.748)	0.607 (0.464, 0.748)	0.637 (0.457, 0.812)
Progression slope (*γ*)	0.035 (0.029, 0.041)	0.028 (0.022, 0.035)	0.038 (0.031, 0.046)
Residual	0.140 (0.130, 0.150)	0.146 (0.136, 0.157)	0.176 (0.162, 0.192)
Thalamus			
Intercept	−3.131 (−3.592, −2.676)	−1.974 (−2.398, −1.53)	−2.556 (−3.142, −1.947)
APOE ε4 carrier	0.002 (−0.132, 0.137)	−0.019 (−0.152, 0.105)	0.012 (−0.131, 0.156)
Progression slope (*γ*)	0.039 (0.033, 0.045)	0.025 (0.019, 0.031)	0.039 (0.031, 0.046)
Residual	0.415 (0.402, 0.427)	0.345 (0.335, 0.356)	0.410 (0.394, 0.427)
ADAS-Cog13			
Intercept	−4.293 (−4.666, −3.939)	−3.206 (−3.569, −2.85)	−7.164 (−7.721, −6.595)
APOE ε4 carrier	0.420 (0.299, 0.539)	0.328 (0.203, 0.449)	0.655 (0.487, 0.832)
Progression slope (*γ*)	0.052 (0.047, 0.057)	0.037 (0.032, 0.042)	0.093 (0.086, 0.101)
Residual	0.367 (0.358, 0.376)	0.391 (0.381, 0.401)	0.334 (0.323, 0.345)
SD of latent time shift (λ)	7.550 (6.041, 8.805)	7.693 (6.219, 9.182)	6.737 (5.734, 7.670)

Values are posterior means with 95% credible intervals (CI). “APOE ε4 carrier” represents the effect of carrying at least one ε4 allele versus non-carriers. “SD of latent time shift (λ)” is the standard deviation of individual latent time shifts (λ_i).

### Quantitative comparison of progression rates and APOE ε4 effects

Table 2 lists the posterior estimates of the progression slope ( $\gamma_{b}$ ) and the APOE ε4 effect for each biomarker and subtype. Key findings: The progression rate of hippocampal volume was fastest in Subtype 3 ( γ=0.096 , 95% CrI 0.089–0.103), compared to 0.064 in Subtype 1 and 0.066 in Subtype 2. The 95% CrI for Subtype 3 did not overlap with those of Subtypes 1 and 2, indicating significantly faster hippocampal atrophy. The slope for ADAS-Cog13 was also steepest in Subtype 3 ( γ=0.093 vs. 0.052 and 0.037). The APOE ε4 effect estimate was largest in Subtype 1: 0.713 (0.603–0.824) for amyloid PET and 0.596 (0.446–0.748) for CSF p-tau. In Subtype 2, the effect estimate was smaller, with CrIs crossing zero for some structural measures. In Subtype 3, the APOE ε4 effect was significant for all biomarkers except thalamus (Table 2), with the largest effect on ADAS-Cog13 (0.655, 95% CrI 0.487–0.832), suggesting that although the genetic risk broadly affects both molecular and structural pathways, its impact on cognitive decline is most pronounced in this rapidly progressing subtype.

To validate the impact of the cortical “N” metric selection on longitudinal modeling results, we conducted a sensitivity analysis by replacing cingulate volume with parietal volume and refitting the latent time model for Subtype 2. The results (Table S2) showed that the two models did not differ significantly in progression slope parameters for any of the six biomarkers (all 95% CIs substantially overlapping), and the temporal ordering of biomarkers remained identical across models, confirming that the “cortex-first, hippocampus-sparing” progression pattern of Subtype 2 is not contingent upon the choice of a specific cortical region.

### Model fit

Scatterplots of observed versus predicted values (Supplementary Figures S3–S5) showed good agreement, indicating appropriate model fit.

### Exploratory external validation in a Chinese cohort

We applied the pre-trained ADNI models to an independent Chinese cohort of 10 patients (Supplementary Table S3). However, predictive performance for thalamic volume and ADAS-Cog13 was more limited, with systematic deviations observed in some individuals (Supplementary Figure S9). These findings suggest that while the core limbic progression pattern generalizes across populations, model performance for other biomarkers may be influenced by cross-ethnic differences in brain structure, genetic background, and clinical assessment practices. Individual-level observed versus predicted values for each Chinese patient are shown in Supplementary Figure S10.

## Discussion

In this study, we integrated SuStaIn subtyping with a latent-time longitudinal joint mixed-effects model to systematically characterise the longitudinal progression of AT(N) biomarkers and cognition across three data-driven AD subtypes in the ADNI cohort. Three main findings emerged. First, the three subtypes differed not only in their spatial order of involvement but also fundamentally in the temporal dynamics of molecular and clinical biomarkers. Second, Subtype 3 exhibited early rapid hippocampal atrophy and strong cognitive coupling, suggesting a ‘subcortical-initiated, limbic-driven’ progression pattern. Third, the effect of APOE ε4 was subtype-dependent: it had the strongest influence on amyloid and tau in the Subtype 1, whereas in Subtype 3 it exerted significant effects on nearly all biomarkers, with the strongest effect on cognition.

Our subtype classification is highly consistent with the literature. Young et al. (2018) first reported three MRI-derived subtypes (typical, cortical, subcortical) using SuStaIn. Murray et al. (2011) described a hippocampus-sparing variant characterised by predominant cortical involvement and younger age of onset. The subcortical-dominant subtype has been less frequently reported, but some studies have noted early striatal amyloid or tau deposition (Cho et al., 2016; Villemagne et al., 2009). The novel contribution of our study lies in quantitatively comparing continuous biomarker trajectories of the three subtypes on a common timescale for the first time, and revealing the rapid hippocampal atrophy feature of Subtype 3 in the longitudinal dimension—a finding that cannot be directly obtained from cross-sectional event sequences.

We note that while the three subtypes in our data show partial overlap with the typical/cortical/subcortical classification of Young et al. (2018)—corresponding broadly to our Subtypes 1, 2, and 3, respectively—in terms of broad spatial patterns, there are non-trivial differences in specific regional event ordering and progression rates: In Subtype 1, the thalamus and nucleus accumbens show early involvement at the earliest stages, which is not fully consistent with the classical Braak staging expectation of exclusive medial temporal lobe onset, suggesting a more complex subcortico-limbic co-initiation pattern. Subtype 2 shows hippocampal slope (0.066) nearly identical to Subtype 1 (0.064), indicating that it is not an extreme “hippocampus-sparing” phenotype but rather a more nuanced “cortico-subcortical” pattern. Therefore, we no longer use prior labels as the primary nomenclature for subtypes in the revised manuscript.

Beyond MRI-based subtyping, recent advances have extended the SuStaIn framework to molecular imaging modalities, offering deeper insights into the upstream pathological drivers of AD. For instance, Vogel et al. applied SuStaIn to tau PET data (Vogel et al., 2021), identifying four distinct trajectories of tau deposition that correlate with differential rates of cognitive decline (Seeley et al., 2009). Similarly, Estarellas et al. developed a missing-data-enabled multimodal SuStaIn extension, integrating structural MRI, amyloid PET, tau PET, and CSF biomarkers to reveal five subtypes that capture both spatial and pathological heterogeneity (Estarellas et al., 2024). These multimodal approaches have the advantage of subtyping based on pathologies more specific to AD, particularly amyloid-β and tau, whereas our MRI-based approach reflects the downstream neurodegenerative consequences of these pathologies. It is important to note that subtypes identified in multimodal studies are not directly equivalent to those defined by atrophy patterns alone; for example, the ‘cortical’ subtype in multimodal frameworks may reflect a different balance of tau and non-AD pathologies (e.g., TDP-43) compared to the Subtype 2 defined strictly by structural loss. Nevertheless, the convergence of evidence across modalities—that AD comprises multiple spatiotemporal progression patterns—strengthens the generalizability of the subtyping concept.

The apparent discrepancy between cross-sectional and longitudinal findings for Subtype 3 deserves further discussion. Cross-sectional SuStaIn detected early subcortical (nucleus accumbens, caudate) *z*-score deviations in this subtype, whereas the longitudinal model showed early rapid hippocampal atrophy. This discrepancy can be reconciled by the network propagation hypothesis: the thalamus and basal ganglia are highly connected hubs, and their early mild volume changes are easily captured as “events” by SuStaIn; however, once pathology spreads to the limbic system (particularly the hippocampus), the subsequent degeneration proceeds extremely rapidly due to the high vulnerability of the hippocampus to tau pathology and neurodegeneration, thereby dominating the longitudinal trajectory (Seeley et al., 2009; Hwang et al., 2017). This “subcortical-initiated, limbic-driven” pattern suggests that cross-sectional event sequences reflect the order of early subtle signals, whereas longitudinal dynamics reveal the key nodes driving disease progression—the two are complementary rather than contradictory.

Notably, Subtype 2 showed earlier thalamic deviation than Subtype 1 (thalamus progression slope (*γ*): 0.025 vs. 0.039 in Subtype 1; Table 2), suggesting that subcortical involvement may coexist with the predominantly cortical involvement pattern. This finding aligns with the network propagation hypothesis (Hwang et al., 2017), where thalamic hubs may facilitate the spread of pathology to cortical regions. However, given the subtle differences between Subtype 1 and Subtype 2 in hippocampal trajectories, the ‘hippocampus-sparing’ label should be interpreted with caution. It is important to distinguish between two parameters: the latent time shift (λ) determines when a biomarker curve begins to deviate along the age axis—the visual observation in Figure 4—while the progression rate (*γ*, i.e., the ‘Progression rate’ column in Table 2) reflects the rate of progression once deviation has begun. The earlier thalamic deviation in Subtype 2 (λ) may reflect early network-level signal propagation through thalamic hubs (Seeley et al., 2009; Hwang et al., 2017), whereas the relatively faster cortical slope (cingulate *γ* = 0.030 vs. thalamus *γ* = 0.025) is consistent with a cortex-prioritized progression pattern. These two parameters are complementary rather than contradictory.

The association of APOE ε4 with biomarker trajectories showed significant heterogeneity across subtypes. In Subtype 1, APOE ε4 was strongly associated with more pronounced amyloid and tau pathology, consistent with the classical APOE-amyloid interaction. In Subtype 2, the effect estimate was smaller and more selective, presumably because the pathological drivers of this subtype may rely more on non-APOE pathways (e.g., TDP-43 or vascular factors). In Subtype 3, APOE ε4 showed significant effect estimates on nearly all biomarkers except thalamus (Table 2), with the largest estimate for cognition (ADAS-Cog13: 0.655, 95% CrI 0.487--0.832). This pattern suggests that although the genetic risk is broadly associated with both molecular and structural pathology, its association with cognitive decline is most pronounced, possibly because this rapidly progressing subtype has already reached a plateau in structural changes, leaving cognitive reserve as the primary dimension in which the genetic effect manifests. These findings suggest that subtype background must be considered when providing genotype-based risk counselling and stratifying clinical trials.

From a methodological perspective, our study demonstrates the advantages of a two-stage analytical framework: first, using SuStaIn for data-driven subtyping to capture spatial heterogeneity, and then performing latent-time joint modelling within each subtype to avoid confounding between spatial and temporal heterogeneity. This “subtype-first, align-second” strategy can be extended to other neurodegenerative diseases (e.g., frontotemporal dementia, Parkinson’s disease) and supports the integration of more modalities of biomarkers (e.g., tau PET, neuroinflammatory markers). At the clinical level, subtype-stratified longitudinal modelling can aid prognostic enrichment and trial design: for example, for the rapidly progressing Subtype 3, clinical trial follow-up duration could be shortened or hippocampal volume could be used as a surrogate endpoint; for the typical subtype where the APOE ε4 effect estimate is largest, ε4 carriers could be preferentially enrolled to enhance power for detecting treatment efficacy.

Several limitations of this study should be acknowledged. First, the ADNI sample is predominantly non-Hispanic white, and external validation in other ethnic groups is required. The results showed that hippocampal volume trajectories, a core structural biomarker, exhibited good cross-population consistency, supporting the generalizability of the limbic-driven progression pattern. However, predictive performance for other biomarkers—particularly thalamus and ADAS-Cog13—was more limited, likely due to cross-ethnic differences in brain structure, genetic background, and clinical assessment practices, as well as the small sample size. We therefore interpret these findings as preliminary evidence rather than definitive validation, and emphasize that larger multi-center Chinese cohorts are needed to fully establish the model’s generalizability across ethnic populations. Second, tau PET or fluid markers of neuroinflammation were not included, limiting direct characterisation of tau spread and inflammatory mechanisms. Third, the model assumes linear progression of each biomarker on the latent time axis; more flexible functions (e.g., sigmoidal) might better capture plateaus. Fourth, sensitivity analyses (e.g., excluding individuals with few visits) were not performed. Fifth, the sample size of Subtype 3 (*n* = 359) was relatively small, which may affect the precision of some estimates, although the posterior credible intervals were within acceptable ranges. Despite these limitations, our study demonstrates that AD subtypes defined by spatial atrophy patterns also differ fundamentally in longitudinal AT(N) biomarker dynamics and APOE ε4 modulation. Subtype-stratified longitudinal modelling provides a more precise framework for disentangling disease heterogeneity and holds promise for improving prognostic prediction and clinical trial design.

## Conclusion

This data-driven analysis based on the ADNI cohort demonstrates that the three AD subtypes (Subtype 1, Subtype 2, and Subtype 3) exhibit significant differences in the longitudinal progression dynamics of AT(N) biomarkers, and that the association of APOE ε4 with disease progression is clearly subtype-dependent. Among these, Subtype 3, although showing early subcortical events in cross-sectional analysis, displays a ‘subcortical-initiated, limbic-driven’ pattern characterised by rapid hippocampal atrophy and tight cognitive coupling in its longitudinal trajectories. The two-stage framework combining SuStaIn subtyping with a latent-time longitudinal mixed-effects model effectively disentangles spatial pathway heterogeneity from temporal rate heterogeneity, providing methodological support for precision subtyping and individualised prognostic assessment. These findings suggest that subtype-specific considerations should be incorporated into clinical trial design and risk stratification to improve statistical power and targeting of interventions.

## Data Availability

Publicly available datasets were analyzed in this study. This data can be found at: Alzheimer’s Disease Neuroimaging Initiative (ADNI), Laboratory of Neuro Imaging (LONI), University of Southern California. Direct link: https://adni.loni.usc.edu/data-samples/access data/. ADNI data are publicly available but require registration and approval of a Data Use Agreement (DUA) before access. Interested researchers can apply for access at the URL provided above.

## Footnotes

1 adni.loni.usc.edu

## Group member of Alzheimer’s Disease Neuroimaging Initiative

Data used in preparation of this article were obtained from the Alzheimer’s Disease Neuroimaging Initiative (ADNI) database (adni.loni.usc.edu). As such, the investigators within the ADNI contributed to the design and implementation of ADNI and/or provided data but did not participate in analysis or writing of this report. A complete listing of ADNI investigators can be found at: https://adni.loni.usc.edu/acknowledgement-list/.

## Glossary

AD: Alzheimer’s disease
ADAS-Cog13: Alzheimer’s Disease Assessment Scale - cognitive subscale (13 items)
ADNI: Alzheimer’s disease neuroimaging initiative
APOE ε4: Apolipoprotein E ε4 allele
AT(N): Amyloid/Tau/Neurodegeneration framework
CI: credible interval
CN: cognitively normal
CrI: credible interval
CSF: cerebrospinal fluid
CVIC: cross-validation information criterion
ECDF: empirical cumulative distribution function
ICV: intracranial volume
MCI: mild cognitive impairment
MCMC: Markov Chain Monte Carlo
MRI: magnetic resonance imaging
PET: positron emission tomography
PVD: positional variance diagram
ROI: region of interest
SD: standard deviation
SuStaIn: subtype and stage inference
