# Supplementary material for: Heterogeneous progression of AT(N) biomarkers in data-driven subtypes of Alzheimer’s disease
Source: Front Aging Neurosci. 2026 Oct 13;18:1902282. doi: 10.3389/fnagi.2026.1902282 (PMC13710405; doi:10.3389/fnagi.2026.1902282)
Supplement: Supplementary file 1 [file Supplementary_file_1.pdf]

Supplementary Material

Table S1. Algorithm parameter settings and subregion-to-lobe mapping for each brain region in the SuStaIn model

Brain region	FreeSurfer region code	FreeSurfer region label	R	Z-sequence	Zmax
Frontal					
	ST15CV	Left Caudal Middle Frontal	3	0.5, 1.5, 2.5	5
	ST25CV	Left Frontal Pole	3	0.5, 1.5, 2.5	5
	ST36CV	Left Lateral Orbitofrontal	3	0.5, 1.5, 2.5	5
	ST39CV	Left Medial Orbitofrontal	3	0.5, 1.5, 2.5	5
	ST55CV	Left Rostral Middle Frontal	3	0.5, 1.5, 2.5	5
	ST56CV	Left Superior Frontal	3	0.5, 1.5, 2.5	5
	ST74CV	Right Caudal Middle Frontal	3	0.5, 1.5, 2.5	5
	ST84CV	Right Frontal Pole	3	0.5, 1.5, 2.5	5
	ST95CV	Right Lateral Orbitofrontal	3	0.5, 1.5, 2.5	5
	ST98CV	Right Medial Orbitofrontal	3	0.5, 1.5, 2.5	5
	ST114CV	Right Rostral Middle Frontal	3	0.5, 1.5, 2.5	5
	ST115CV	Right Superior Frontal	3	0.5, 1.5, 2.5	5
Temporal					
	ST32CV	Left Inferior Temporal	3	0.5, 1.5, 2.5	5
	ST40CV	Left Middle Temporal	3	0.5, 1.5, 2.5	5
	ST58CV	Left Superior Temporal	3	0.5, 1.5, 2.5	5
	ST60CV	Left Temporal Pole	3	0.5, 1.5, 2.5	5
	ST62CV	Left Transverse Temporal	3	0.5, 1.5, 2.5	5
	ST91CV	Right Inferior Temporal	3	0.5, 1.5, 2.5	5
	ST99CV	Right Middle Temporal	3	0.5, 1.5, 2.5	5
	ST117CV	Right Superior Temporal	3	0.5, 1.5, 2.5	5
	ST119CV	Right Temporal Pole	3	0.5, 1.5, 2.5	5
	ST121CV	Right Transverse Temporal	3	0.5, 1.5, 2.5	5
Parietal					
	ST31CV	Left Inferior Parietal	3	0.5, 1.5, 2.5	5
	ST57CV	Left Superior Parietal	3	0.5, 1.5, 2.5	5
	ST90CV	Right Inferior Parietal	3	0.5, 1.5, 2.5	5
	ST116CV	Right Superior Parietal	3	0.5, 1.5, 2.5	5
Occipital					
	ST35CV	Left Lateral Occipital	3	0.5, 1.5, 2.5	5
	ST94CV	Right Lateral Occipital	3	0.5, 1.5, 2.5	5

Cingulate					
	ST14CV	Left Caudal Anterior Cingulate	3	0.5, 1.5, 2.5	5
	ST34CV	Left Posterior Cingulate	3	0.5, 1.5, 2.5	5
	ST50CV	Left Isthmus Cingulate	3	0.5, 1.5, 2.5	5
	ST54CV	Left Rostral Anterior Cingulate	3	0.5, 1.5, 2.5	5
	ST73CV	Right Caudal Anterior Cingulate	3	0.5, 1.5, 2.5	5
	ST93CV	Right Posterior Cingulate	3	0.5, 1.5, 2.5	5
	ST109CV	Right Isthmus Cingulate	3	0.5, 1.5, 2.5	5
	ST113CV	Right Rostral Anterior Cingulate	3	0.5, 1.5, 2.5	5
Insula					
	ST129CV	Left Insula	3	0.5, 1.5, 2.5	5
	ST130CV	Right Insula	3	0.5, 1.5, 2.5	5
Hippocampus					
	ST29SV	Left Hippocampus	3	1.0, 2.0, 3.0	5
	ST88SV	Right Hippocampus	3	1.0, 2.0, 3.0	5
Amygdala					
	ST12SV	Left Amygdala	3	1.0, 2.0, 3.0	5
	ST71SV	Right Amygdala	3	1.0, 2.0, 3.0	5
Thalamus					
	ST61SV	Left Thalamus	3	0.5, 1.5, 2.5	5
	ST120SV	Right Thalamus	3	0.5, 1.5, 2.5	5
Accumbens					
	ST11SV	Left Accumbens	3	0.5, 1.5, 2.5	5
	ST70SV	Right Accumbens	3	0.5, 1.5, 2.5	5
Caudate					
	ST16SV	Left Caudate	3	0.5, 1.5, 2.5	5
	ST75SV	Right Caudate	3	0.5, 1.5, 2.5	5

Table S2. Sensitivity analysis for cortical marker selection in Subtype 2: comparison of progression slope effects between cingulate and parietal models

Biomarker	Cingulate Model (95% CI)	Parietal Model (95% CI)
Hippocampus (progression slope)	0.066 (0.062, 0.071)	0.064 (0.059, 0.069)
Cortical marker (progression slope)	0.030 (0.025, 0.036)	0.031 (0.025, 0.038)
ADAS-Cog13 (progression slope)	0.037 (0.032, 0.042)	0.039 (0.033, 0.044)
Amyloid PET (progression slope)	0.028 (0.023, 0.033)	0.027 (0.022, 0.032)

CSF p-tau (progression slope)	0.028 (0.022, 0.035)	0.029 (0.023, 0.036)
Thalamus (progression slope)	0.025 (0.019, 0.031)	0.024 (0.018, 0.030)

Notes: progression slope (γ) represent the rate of change per unit latent time for each biomarker in Subtype 2. Overlapping 95% CIs across models confirm that findings are robust to the choice of cortical ROI (cingulate vs. parietal).

Table S3 Demographic characteristics, subtype assignment, and estimated latent time shifts of Chinese patients in the external validation cohort.

ID	Age	Sex	APOE	Diagnosis	Subtype	Latent time shift (λ_i)
001	74	F	ε3/ε3	AD	2	3.36631
002	76	F	ε3/ε3	Cognitive impairment	3	-0.33118
003	56	F	ε3/ε3	Cognitive impairment	3	10.66294
004	79	M	ε3/ε4	Cognitive impairment	1	0.772526
005	80	M	ε4/ε4	AD	1	7.537513
006	62	F	ε2/ε4	AD	2	1.568149
007	79	M	ε4/ε4	Cognitive impairment	2	3.207107
008	64	M	ε3/ε4	Cognitive impairment	1	6.855426
009	62	M	ε3/ε4	Cognitive impairment	1	3.496366
010	63	M	ε3/ε3	Cognitive impairment	2	1.404651

AD, Alzheimer's disease; APOE, apolipoprotein E.

Table S4. Sample size of the six longitudinal biomarkers stratified by baseline

diagnosis.

Baseline diagnosis	N
Cognitively normal	639
Amyloid PET	496
CSF p-tau	672
ADAS-Cog13	674
Hippocampus	
Mild cognitive impairment	631
Amyloid PET	540
CSF p-tau	687
ADAS-Cog13	179

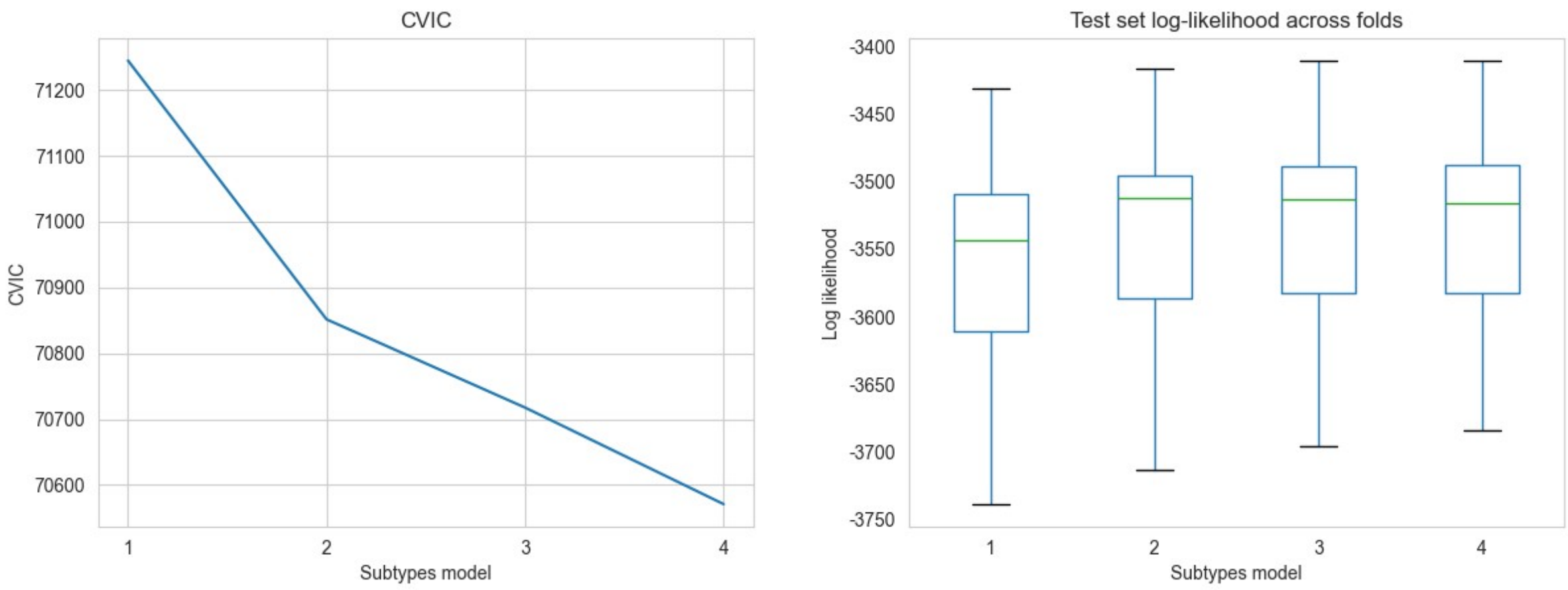

Figure S1. Model selection for SuStaIn subtype number.

(A) Cross-validation information criterion (CVIC) values for models with 1 to 4 subtypes. (B) Average test set log-likelihood values. Both criteria show that the three-subtype model provides an optimal balance between model fit and complexity, with diminishing improvements beyond three subtypes. CVIC: 1-subtype = 71243.97, 2-subtype = 70851.39, 3-subtype = 70717.53, 4-subtype = 70571.05.

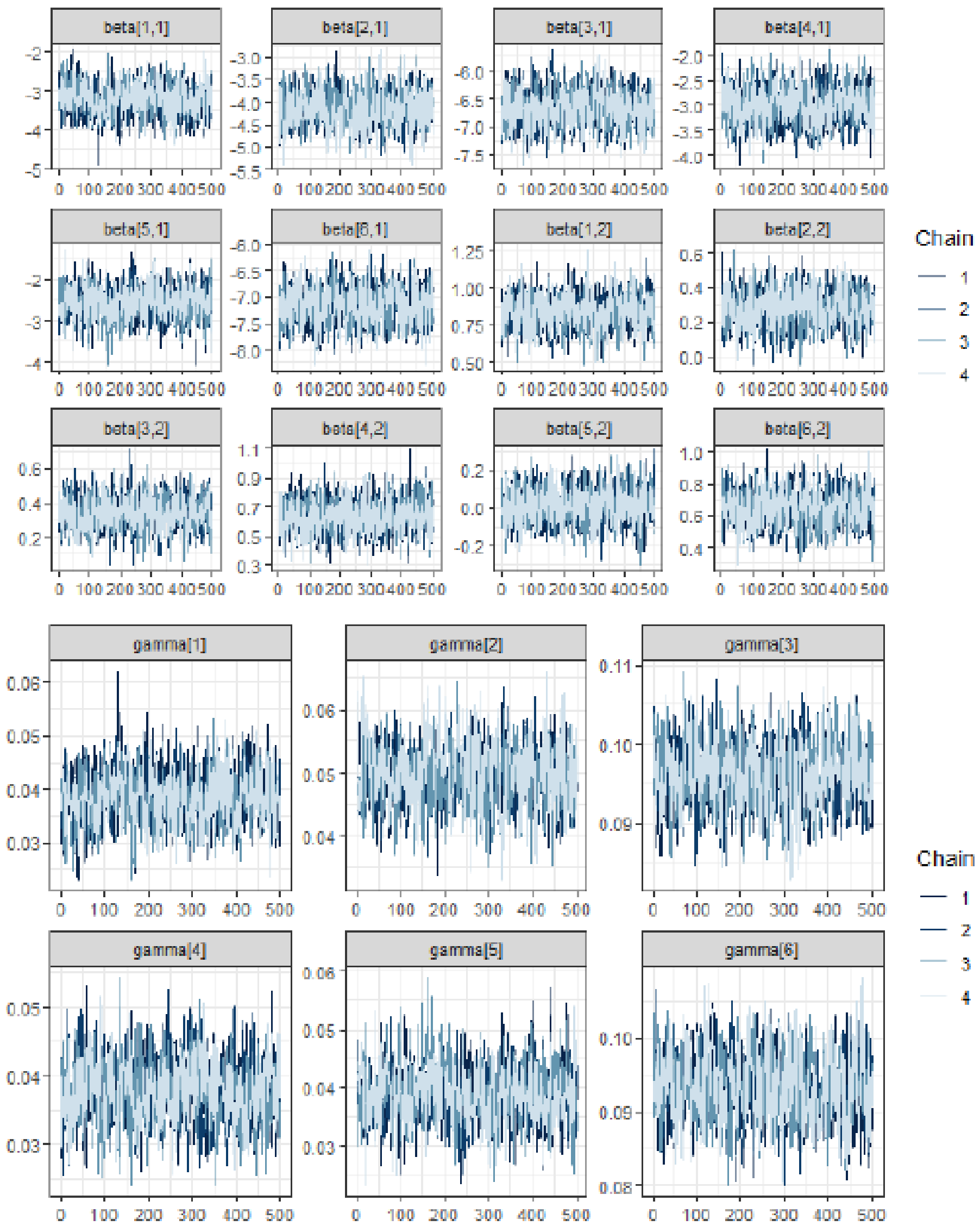

Figure S2. Trace plots of selected parameters for Subtype 3.

Four chains show good mixing after warm-up, confirming convergence ($\hat{R} < 1.05$ for all parameters).

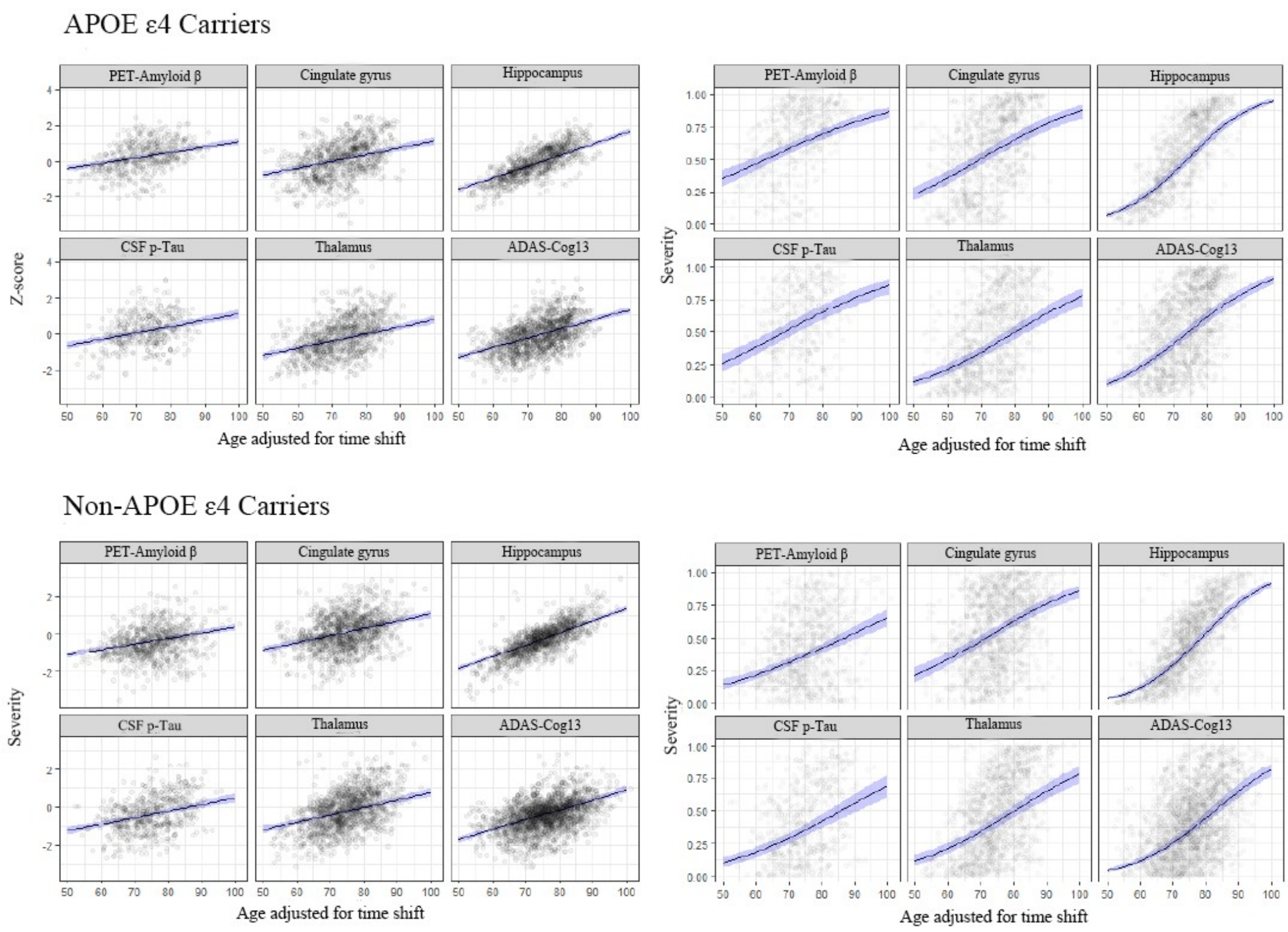

Figure S3. Scatter plots of observed versus predicted values for Subtype 1.

Each panel represents one of the six biomarkers (amyloid PET, CSF p-tau, hippocampal volume, cingulate volume, thalamus volume, ADAS-Cog13). Points cluster around the diagonal line, indicating good model fit. A non-APOE-stratified version of this figure is provided in Figure S8.

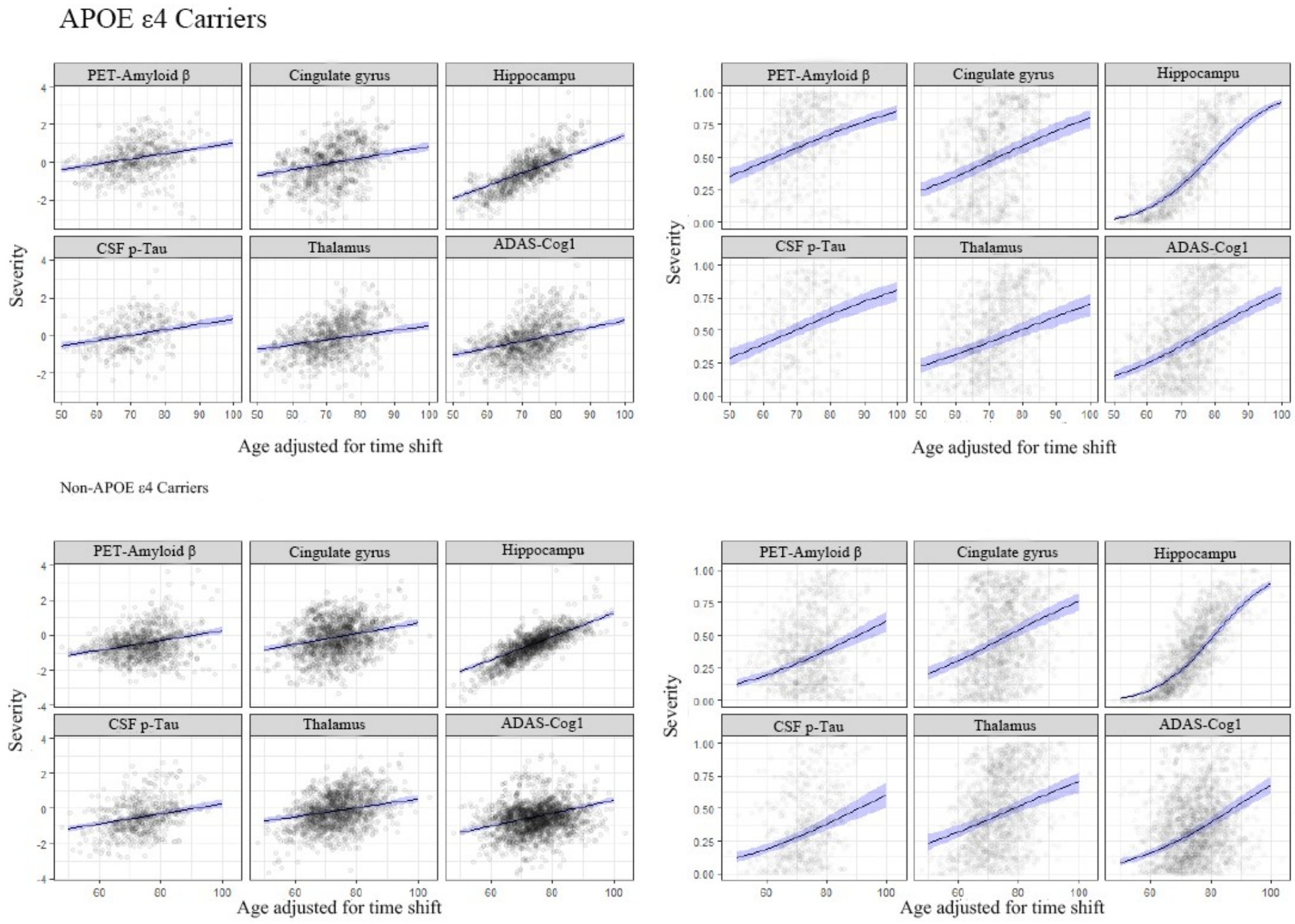

Figure S4. Scatter plots of observed versus predicted values for Subtype 2.

Each panel represents one of the six biomarkers (amyloid PET, CSF p-tau, hippocampal volume, cingulate volume, thalamus volume, ADAS-Cog13). The solid diagonal line indicates perfect agreement (y = x), where predicted values equal observed values. Points clustering around this line indicate good model fit. This figure corresponds to the model fit evaluation described in Section 3.6 and complements Figures S3 and S5 for Subtypes 1 and 3, respectively.

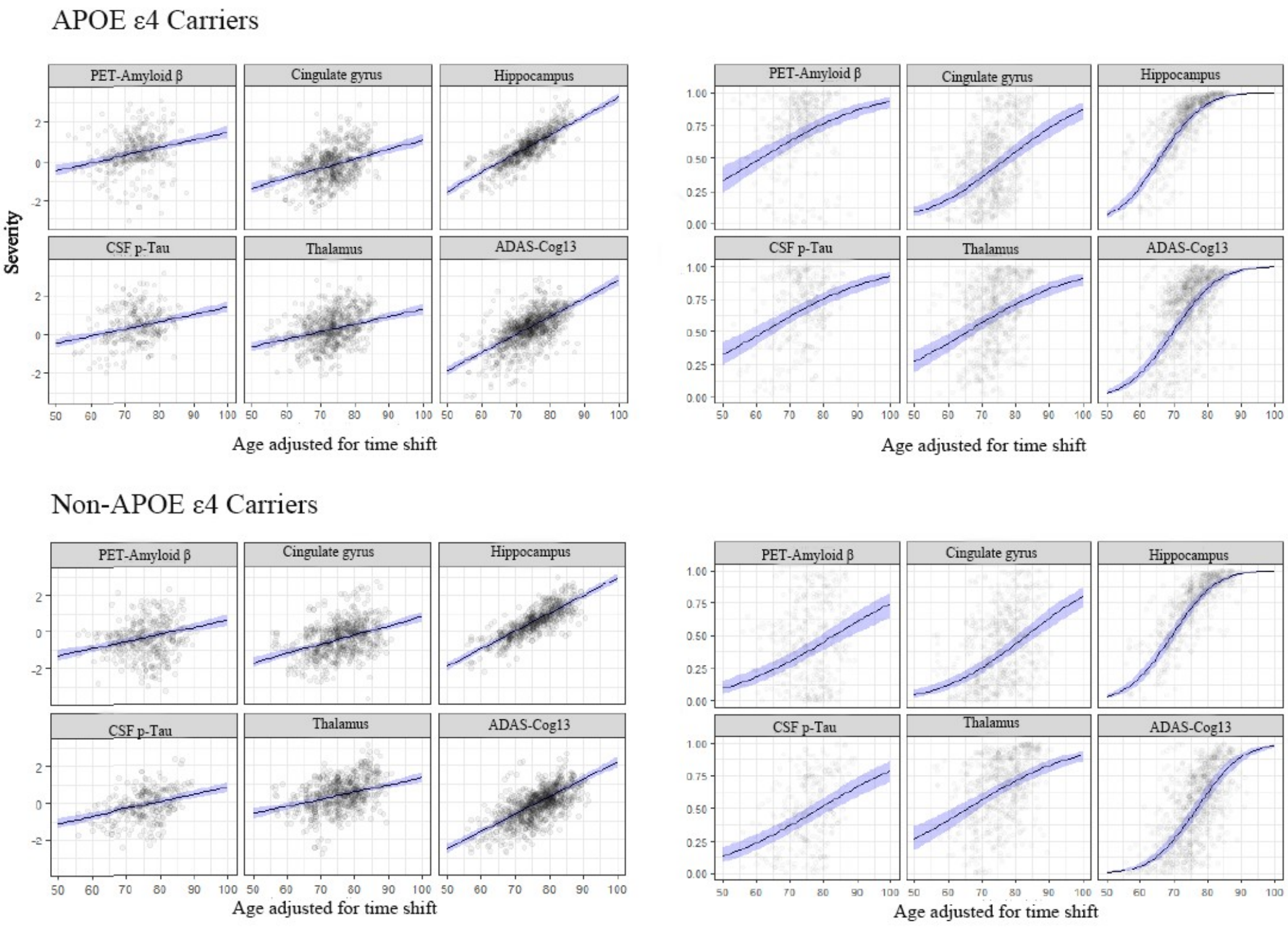

Figure S5. Scatter plots of observed versus predicted values for Subtype 3.

Each panel represents one of the six biomarkers (amyloid PET, CSF p-tau, hippocampal volume, cingulate volume, thalamus volume, ADAS-Cog13). Points cluster around the diagonal line, indicating good model fit for the Subtype 3.

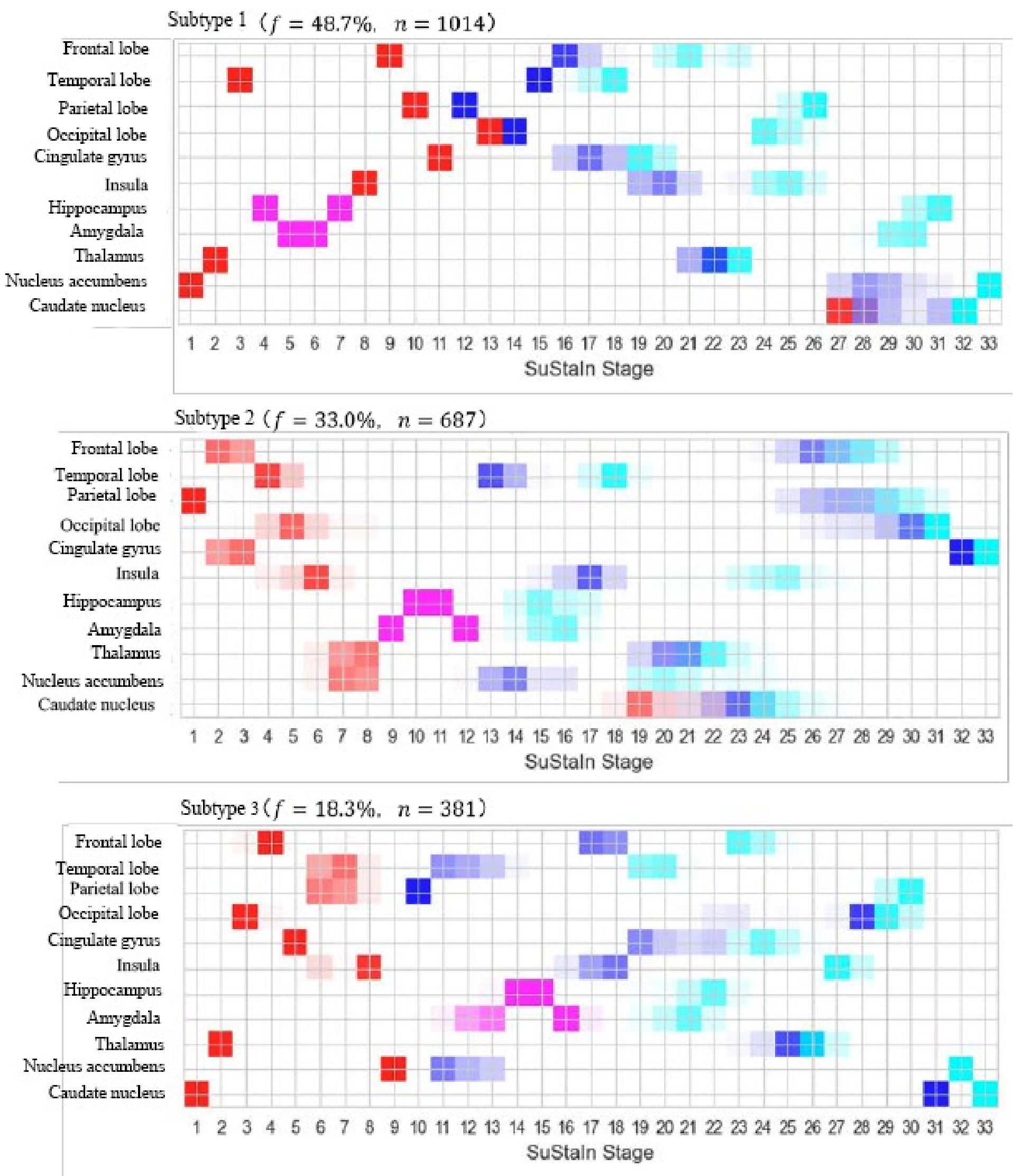

Figure S6. Positional variance diagram (PVD) from pooled longitudinal data.

Subtype structures are consistent with cross-sectional results (Figure 1). Color intensity represents posterior probability of event occurrence at each stage.

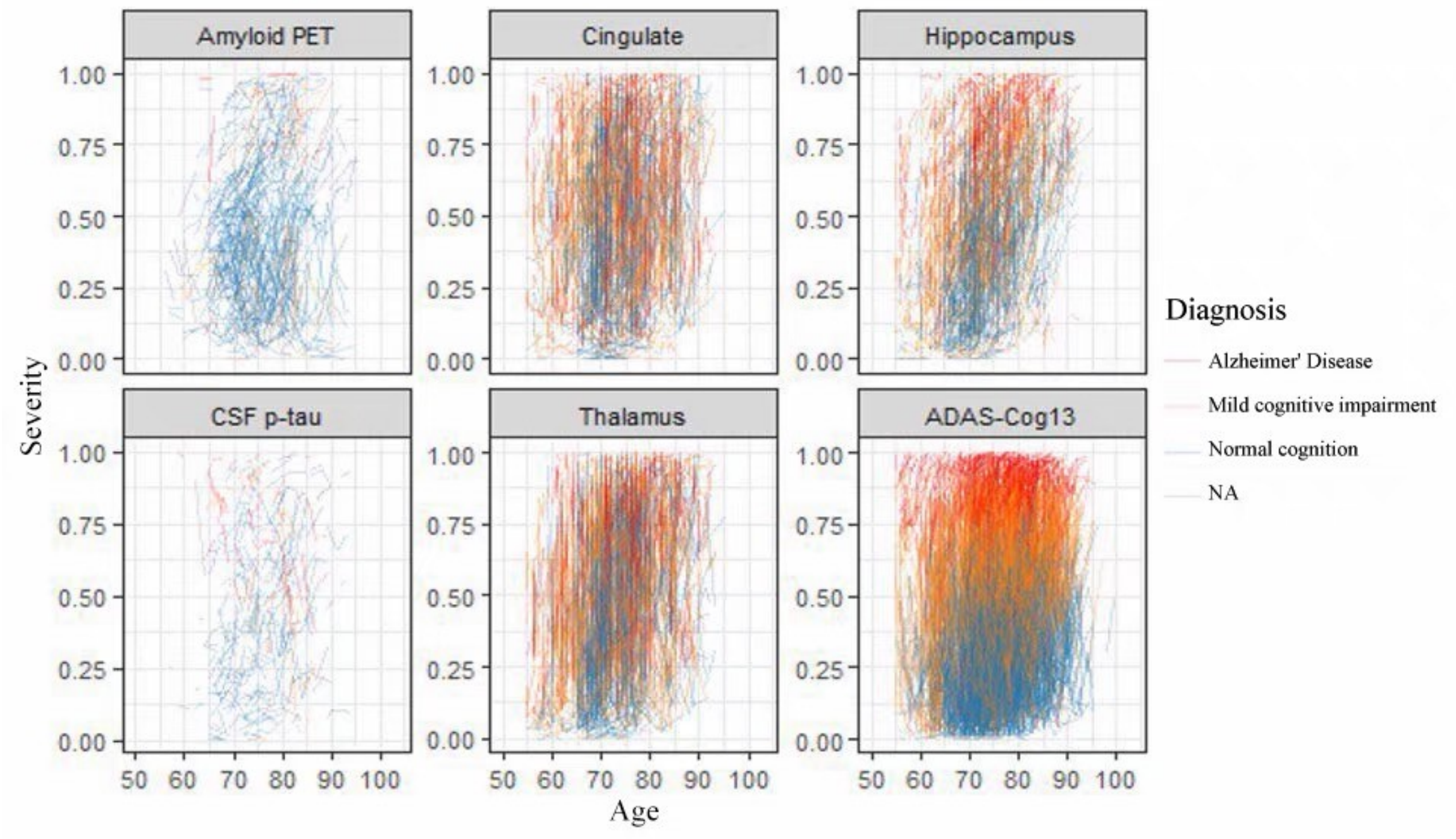

Figure S7. Uncorrected age trajectories of AT(N) biomarkers by diagnostic group.

These trajectories show the raw age-related changes of the six biomarkers (amyloid PET, CSF p-tau, hippocampal volume, cingulate volume, thalamic volume, and ADAS-Cog13) prior to latent time shift correction. Lines are color-coded by clinical diagnosis: blue = cognitively normal (CN), orange = mild cognitive impairment (MCI), red = Alzheimer's disease (AD). Without latent time alignment, the trajectories across diagnostic groups exhibit considerable overlap and do not follow a clear monotonic order from CN to MCI to AD. This figure provides the baseline comparison for Figure 3 in the main text, which shows the same biomarkers after latent time shift correction, where the ordering of trajectories becomes clearly monotonic across diagnostic groups. ADAS-Cog13, Alzheimer's Disease Assessment Scale-Cognitive Subscale (13 items).

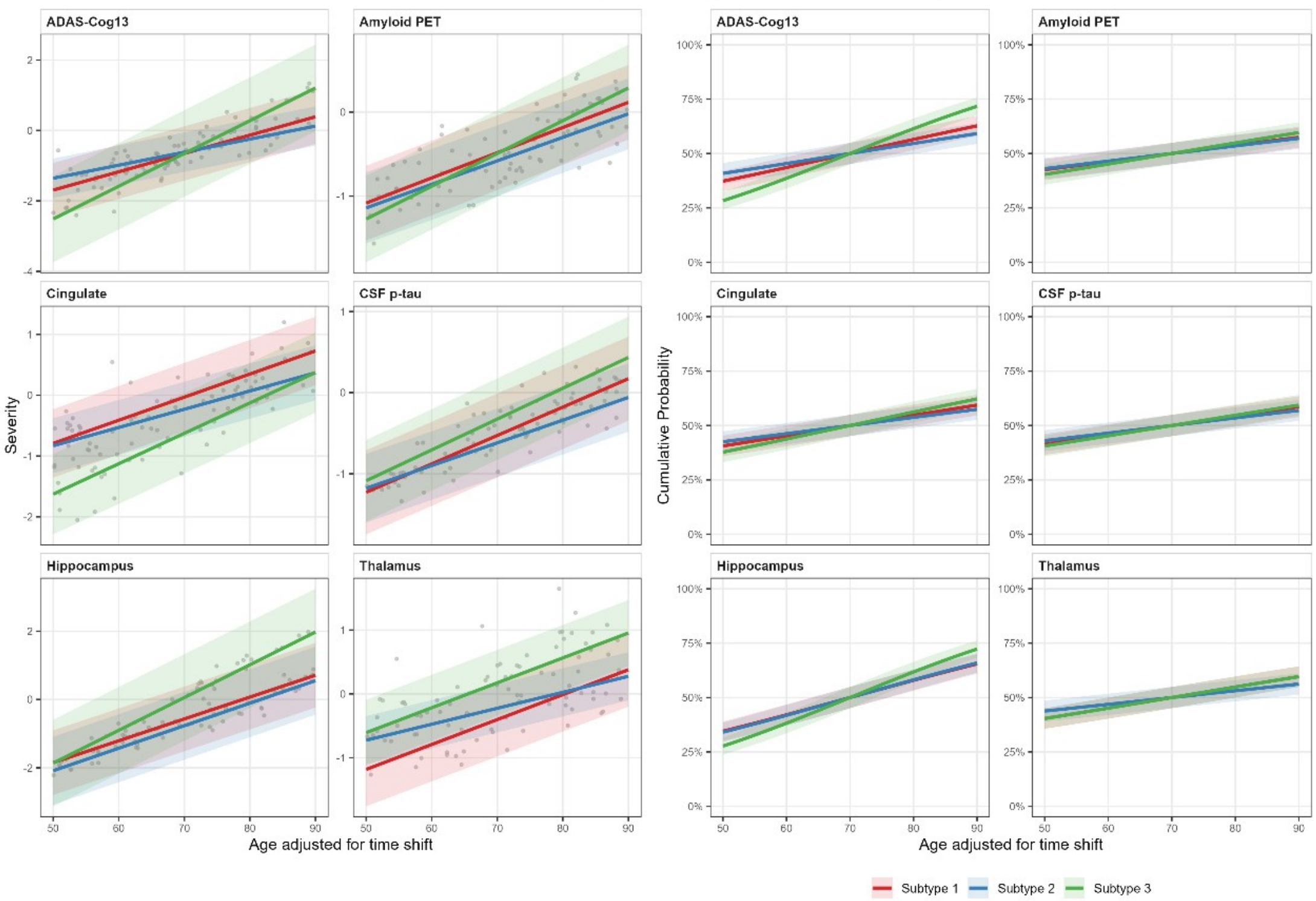

Figure S8 Non-APOE-stratified longitudinal trajectories of AT(N) biomarkers across subtypes

Colors represent the three data-driven subtypes (red: Subtype 1; blue: Subtype 2; green: Subtype 3). Left panels: Gray points represent individual-level observations, solid lines show fitted trajectories, and shaded bands denote 95% confidence intervals. Right panels: Curves represent the cumulative proportion of each subtype reaching a given severity level over time. X-axis represents age adjusted for latent time shift.

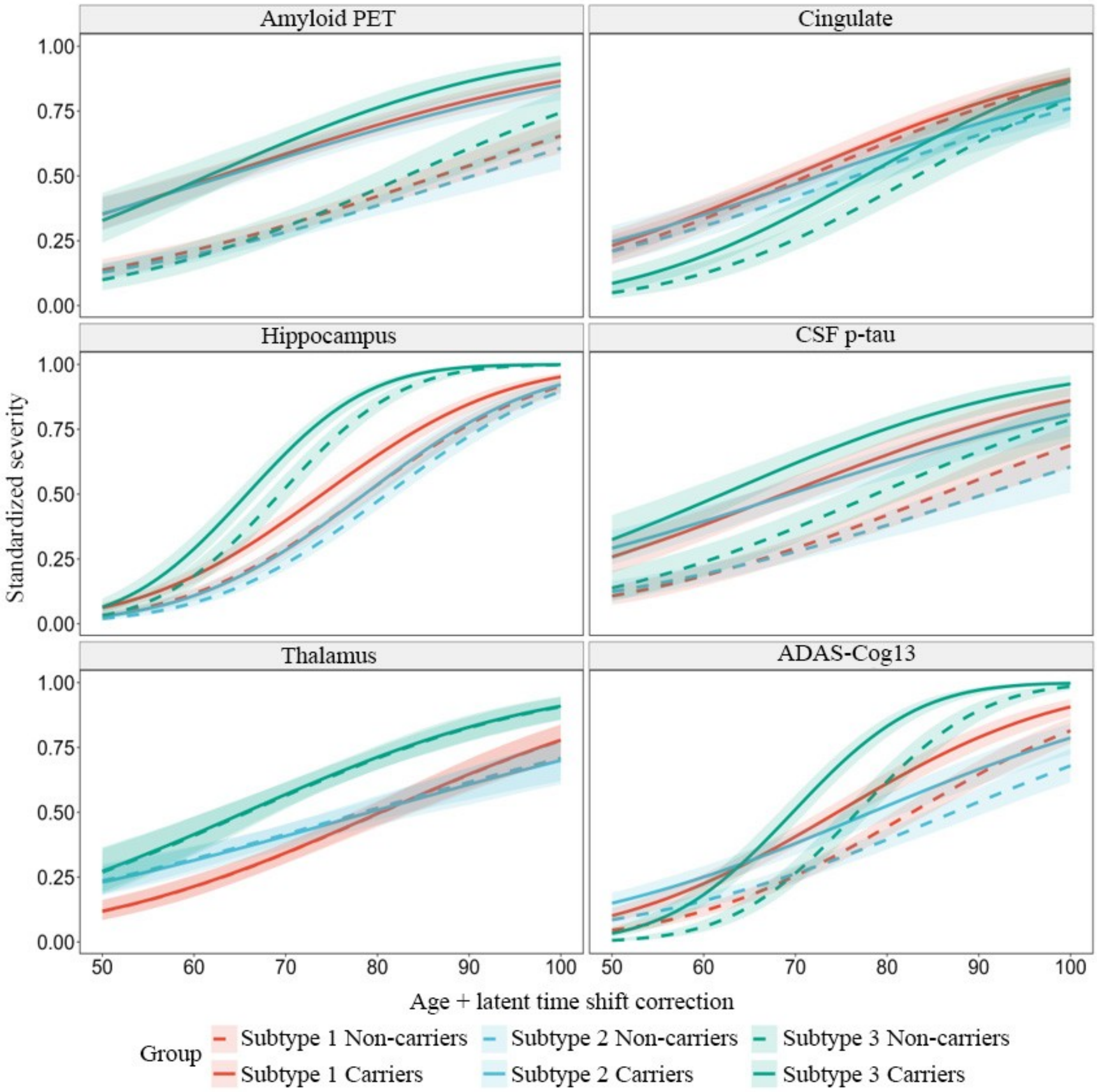

Figure S9 Trajectory differences of biomarkers across subtypes over disease time.

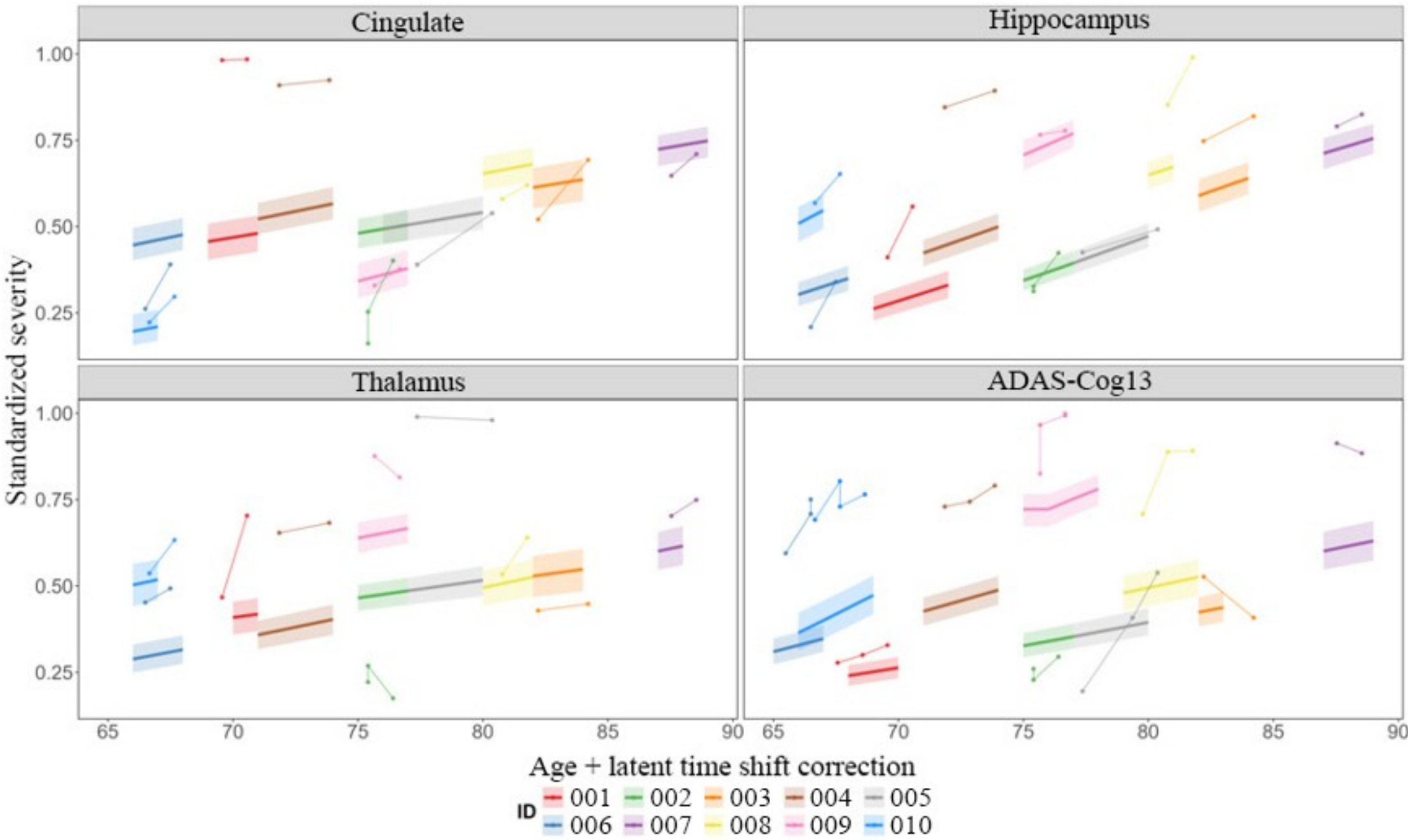

Figure S10 Observed versus predicted biomarker trajectories for 10 Chinese patients in the external validation cohort.
